# Effective Implementation Strategies for Delivering Nutritional Interventions through the Health System to Prevent Malnutrition during Pregnancy: A Systematic Review and Meta-analysis

**DOI:** 10.1016/j.advnut.2026.100642

**Published:** 2026-04-29

**Authors:** Selene Pacheco-Miranda, Edith Kim-Herrera, Rafael Peréz-Escamilla, Edgar Denova-Gutiérrez, Hortensia Reyes-Morales, Lucía Hernández-Barrera, Enrique Rios-Espinosa, Alejandra Gonzalez-Rocha, Anabelle Bonvecchio-Arenas

**Affiliations:** 1 School of Public Health, National Institute of Public Health of Mexico, Cuernavaca, Mexico; 2 Nutrition and Health Research Center, National Institute of Public Health, Cuernavaca, Mexico; 3 Department of Social and Behavioral Sciences, Yale School of Public Health, New Haven, CT, United States; 4 Department of Nutrition, National Institute of Medical Sciences and Nutrition Salvador Zubirán, Mexico City, Mexico; 5 Health System Research Center, National Institute of Public Health, Cuernavaca, Mexico; 6 Vitamin Angels Alliance, Goleta, CA, United States; 7 Social Interventions Research & Evaluation Network, University of California, San Francisco, CA, United States

**Keywords:** nutritional interventions, pregnancy, implementation strategies, malnutrition, health system, primary health care

## Abstract

Malnutrition during pregnancy is a public health concern. Interventions implemented through the health sector can prevent maternal malnutrition. Our aim was to identify implementation strategies for delivering nutritional interventions through primary health care to prevent malnutrition in all its forms during pregnancy. We followed the *Cochrane Handbook for Systematic Reviews* and PRISMA guidelines. A search strategy was developed for 5 databases. The information was systematized using the Template for Intervention Description and Replication. Meta-analyses were performed using a random-effects model. We used the Risk of Bias (RoB) and the Nonrandomized Studies of Interventions tools, and the certainty of the evidence followed the Grading of Recommendations, Assessment, Development, and Evaluation guidelines. We included 51 studies conducted across high-, middle-, and low-income countries. Multiple micronutrient supplementation (MMS) was more effective than iron and folic acid (IFA) supplementation alone in improving hemoglobin concentrations and other anemia-related indicators, when initiated during the first or second trimester of pregnancy and delivered with in-person, individualized counseling and follow-up. Our meta-analysis confirmed that MMS improved maternal anemia compared with IFA with a moderate certainty of the evidence. Healthy eating counseling, physical activity, and weight-gain monitoring, when combined, were effective in achieving weight gain when the interventions were guided by prenatal care protocols and included materials and resources to support. Our meta-analysis showed a nonsignificant reduction in excessive weight gain with very low certainty, no meaningful effect on low weight gain with low certainty, and a potentially meaningful increase in the likelihood of gaining weight within the recommended range with very low certainty. A coordinated package of health system–delivered interventions, including MMS, behavioral counseling, and monitoring of maternal weight gain, should be implemented within primary health care, beginning in the first or second trimester, to prevent all forms of malnutrition during pregnancy. Effective implementation strategies to provide these interventions can be adapted to local contexts.

The protocol for this review was registered at PROSPERO as CRD 4202460299.

**Statement of Significance**

This systematic review and meta-analysis provides guidance on effective ways to implement maternal nutrition interventions, enabling their adoption and adaptation to the specific context of each country, while accounting for its structure, multisectoral coordination, and financing.

## Introduction

Malnutrition is a significant public health concern defined by deficiencies, excesses, or imbalances in an individual’s nutrient or calorie intake [1]. Pregnant women are particularly vulnerable to this condition in all its forms [1,2]. Globally, 6.9 million pregnant women (25%) are undernourished [1]. Also, anemia remains highly prevalent, affecting 36.5% of pregnant women in 2022, with iron deficiency as the leading cause [3,4]. In addition, worldwide, 53.9% of pregnant women [5] experience inadequate weight gain, 22% gain excessive weight, whereas ∼38 million live with overweight, and 14.6 million live with obesity [6].

Malnutrition during pregnancy increases the risk of maternal and fetal mortality, labor and delivery complications, and metabolic disorders. It is also linked to fetal growth restriction, low birth weight, and long-term health risks for the offspring, including obesity and chronic diseases, impacting economic productivity and health care costs. Obesity in women can negatively affect breastfeeding and promote infant formula use [[7], [8], [9], [10], [11]]. It is essential to highlight that malnutrition during pregnancy, whether due to deficiencies or excesses, has lasting effects in the early years of the offspring’s life. It contributes to an imbalance in the gut microbiome, promotes inflammation and metabolic dysregulation, and alters insulin signaling, thereby favoring the development of obesity and other noncommunicable diseases in later life, particularly in adulthood [1,12].

The health system is considered the leading platform for implementing nutritional interventions aimed at preventing malnutrition in pregnant women [[13], [14], [15]]. These interventions include micronutrient supplementation, monitoring of weight gain during pregnancy, and counseling to promote healthy eating and encourage physical activity [14].

Integrating evidence-based nutritional interventions into health system policies and programs, particularly through maternal health services, can significantly benefit pregnant women and be a cost-effective approach. To promote health equity, these interventions should prioritize access for marginalized populations and be integrated into universal health coverage, ensuring all women have access to them for improved health and reduced health care costs [2,13,16].

In this regard, the global guidelines for implementing these interventions are primarily based on evidence gathered from low- and middle-income countries for micronutrient supplementation. In contrast, guidelines for healthy eating and physical activity were derived from high-income countries. Therefore, they emphasize populations with overweight and obesity, whereas, in middle- and low-income countries, the coexistence of different forms of malnutrition among pregnant women is increasingly observed [17].

In addition, various studies conducted in low- and middle-income countries have documented that these guidelines lack detailed implementation instructions for the delivery of nutritional interventions to pregnant women at the primary health care level. This is of concern because standard operating procedures are needed for defining the ideal profile of providers, the appropriate dosage, the intervention delivery method (e.g., individual, group, or both), and whether these interventions should be provided face-to-face or in a virtual format, as well as the necessary resources to promote behavior change among the target population [18].

It has been reported that these interventions have not been adequately adapted to the nutritional needs or the sociocultural and economic context of pregnant women, and do not consider some essential aspects of the primary health care context, such as its operational or organizational structure, which could limit the proper delivery, sustainability, and reach of these interventions and their integration into the health system [19,20].

Therefore, this paper aimed to identify the most effective implementation strategies, that is, the specific forms [21] for delivering nutritional interventions through the primary health care to prevent malnutrition in all its forms during pregnancy, to improve their integration into the health system, and to ensure that no pregnant woman is deprived of her right and opportunity to receive the comprehensive care that enables her to have a healthy pregnancy.

## Methods

This review followed the methodology outlined in the *Cochrane Handbook for Systematic Reviews of Interventions* [22] and was reported in accordance with the PRISMA guidelines [23]. The protocol for this review was registered in PROSPERO with the ID CRD42024602991.

### Inclusion and exclusion criteria

#### Type of studies

We included randomized controlled trials (RCTs), cohort studies, quasi-experimental studies, and case-control studies from their inception in the database up to January 2025, without language restrictions. We excluded all types of reviews, guidelines, commentaries, qualitative studies, and letters to the editor.

#### Types of participants

Studies including healthy pregnant women of any age and health care providers within primary health care services were considered. Studies involving women with a prior diagnosis of preeclampsia, eclampsia, gestational diabetes, anemia, overweight, obesity, or other health comorbidities were excluded because the focus was on identifying interventions aimed at preventing various forms of malnutrition, implemented in typical real-world settings where women are generally unaware of their nutritional status and have not yet received specific treatment. Interventions for inpatients and clinical trials conducted outside the primary health care context (secondary and tertiary care hospitals, university research centers, or other platforms outside primary health care) were also excluded.

#### Types of interventions

Studies involving the nutritional interventions of interest included supplementation with multiple micronutrients containing iron and folic acid (IFA), gestational weight-gain monitoring, dietary counseling, and promotion of physical activity. We excluded studies focused solely on iron or folic acid (FA) supplementation, given prior evidence that multiple micronutrient supplementation (MMS) yields greater maternal and child health benefits [24]. Balanced energy and protein supplements were also excluded, given that although some of them contain multiple micronutrients, they are intended explicitly for undernourished populations [17].

#### Outcomes

We included studies reporting outcomes related to different forms of malnutrition among pregnant women, such as hemoglobin concentrations and the percentage of abnormal hemoglobin; anemia-related indicators (e.g., prevalence or ferritin and serum transferrin receptor concentrations); changes in mid-upper arm circumference (MUAC) in centimeters to assess acute malnutrition; BMI; gestational weight gain categorized as total, low/inadequate, healthy/adequate, or high/excessive based on the Institute of Medicine (IOM) recommendations; weight at delivery, among others. Studies focusing exclusively on child outcomes were also excluded.

### Electronic searches

The search strategies were developed using the Population, Intervention, Control/Comparison group, and Outcomes (PICOS) model for each intervention. The databases searched were EMBASE, Science Direct, Web of Science, Cochrane Reviews, and PubMed. Validated Medical Subject Headings (MeSH) terms, along with additional terms agreed upon through expert consensus (SP-M, EK-H, LH-B, and AB-A) (e.g., “pregnancy,” “multiple micronutrients,” “health assessment,” “physical activity,” “nutrition,” “primary care,” “antenatal,” “prenatal,” and “health system”), were used. The detailed search strategy for each database is provided in Supplementary Table 1.

### Selection of studies

**FIGURE 1:**
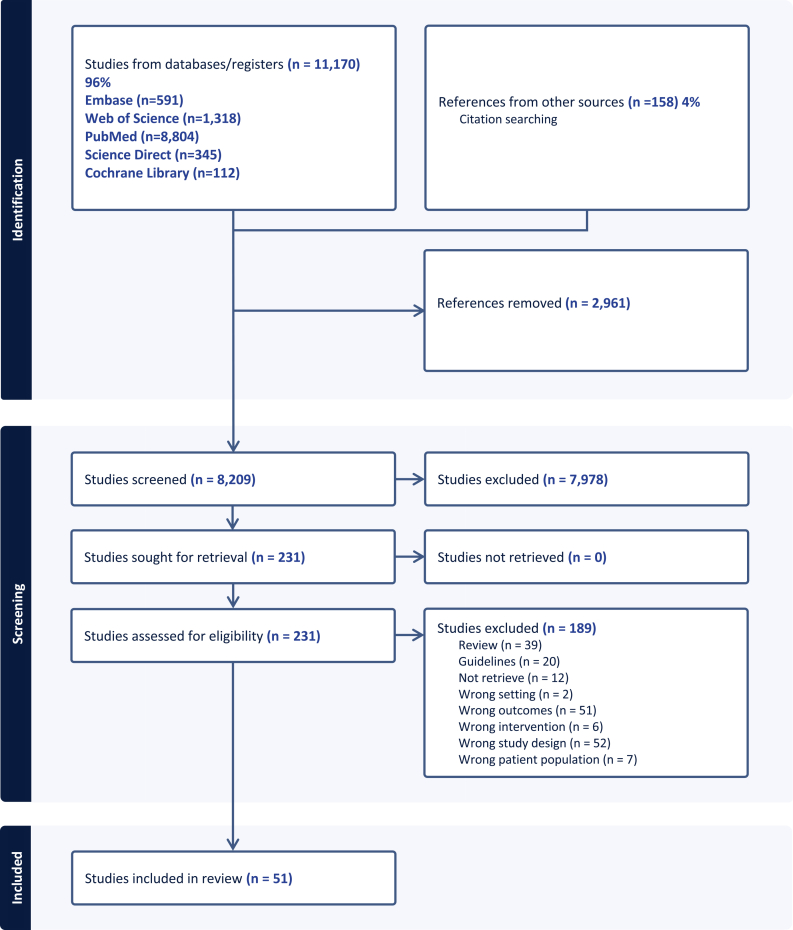
Flow chart of the selection process for the included studies in the systematic review of effective implementation strategies for delivering nutritional interventions to prevent malnutrition during pregnancy.

The systematization and organization of articles were carried out using the Covidence platform. Titles and abstracts were screened independently (SP-M, AG-R, and EK-H), and duplicate reviews were conducted to identify relevant studies; those that did not meet the inclusion criteria were removed. Also, full texts were retrieved and systematically examined for inclusion or exclusion criteria in duplicate by the same team. In the event of disagreement, another author (ED-G) assisted in reaching a final decision. The flowchart of the selection process is shown in Figure 1.

### Other sources

Following a snowball approach, 2 authors (AG-R and SP-M) scanned the titles of the references of previous systematic reviews similar to ours and within the included studies. All the relevant titles identified were added to the Covidence software, which automatically deleted duplicates.

### Data extraction and management

The extracted form was created in accordance with the PICOS model. We identified the initial characteristics of the population, as well as the type of intervention and comparison. An adaptation of the Template for the Description and Replication of Interventions (TIDieR) framework was used to guide the systematization of the information [25]. We characterized the implementation strategies of interventions and their key elements, including the necessary resources or materials, provider profiles, modes or formats of delivery, locations, frequencies, and other associated factors (Table 1).

**TABLE 1:** Key implementation elements considered for the systematization of the collected information in the systematic review and adapted from the TIDieR framework^26^.

Item	Description	Key elements for implementation
Why?	Includes any model or guide used for the design of the intervention.	Based on a guideline: if the intervention or set of interventions was based on international recommendations, guidelines, or protocols.
		Based on a model: if the intervention or set of interventions was based on a behavior change model.
What?	Involves materials to deliver the intervention, including those provided to participants.	Resources or materials used for delivering the intervention: brochures, recipes, videos, text messages, phone calls, manuals, applications, and websites, among others.
Provider	Person responsible for implementing the intervention.	Doctor, nurse, midwife, nutritionist, community health worker, others.
In what way?	Describe the delivery methods (e.g., face-to-face or through other mechanisms, such as the internet or phone) of the intervention and whether it was provided individually or in a group.	Delivery method: individual/virtual or mix.
		How? Face-to-face, virtually (via phone, text messages, virtual platform), or a mix.
Where?	Describe the type or types of platforms where the intervention was carried out.	Antenatal clinics: antenatal centers, prenatal care centers or clinics, metropolitan care facilities, outpatient clinics, and obstetric clinics. Health centers: primary health units. Community clinics: village, rural, or community hospitals with primary health care services. Home visits: with the support of health personnel. Community recreation.
When?	Time when the intervention began to be implemented with the participants.	Pregnancy trimester: 1st, 2nd, 3rd.
Frequency	It refers to the delivery frequency by the intervention implementers.	Biweekly (twice a week), weekly, monthly, or in routine appointments (specifying the number of consultations).
Dose	It refers to the amount of an intervention that is delivered or received by the target participants.	For supplementation-related interventions, the recommended dose of the supplement was described to the participants. For the remaining interventions, the time spent in each contact with the staff providing them was documented.
Duration	The duration of supplementation, counseling, or actions to monitor weight gain in pregnant women was described.	Until delivery or for weeks (e.g., 12) or months postpartum (e.g., 6 mo).
Tailoring	If the intervention was planned to be personalized, adjusted, or adapted to the sociocultural determinants of the participants.	Sociocultural context of the target population, for example: translation of materials or sessions into the local language, acknowledgment of religious and cultural aspects in intervention development, counseling on nutrition based on local availability, and identifying the needs and concerns of the women.

Abbreviation: TIDieR, Template for Intervention Description and Replication.

In addition, we extracted the mean difference, SD, confidence interval (CI), odds ratio (OR), and prevalence for the different forms of malnutrition established in the inclusion criteria, when available. In cases where information was missing, we contacted the first and corresponding authors of the included articles.

#### Meta-analysis

We performed a pooled analysis for studies with comparable data. When the measures presented were mean difference and SD or CI, we used the inverse variance statistical method. When the OR or prevalence of the condition with SD or CI was given, we used the Cochran–Mantel–Haenszel test, both with a random-effects model. The heterogeneity estimator was calculated with the method of “Restricted Maximum Likelihood,” and *I*^2^ was presented [24]. *I*^2^ was interpreted: 0% to 40%: might not be important, 30% to 60%: may represent moderate heterogeneity, 50% to 90%: may represent substantial heterogeneity, 75% to 100%: considerable heterogeneity. We performed leave-one-out analysis and sensitivity analysis, removing high-income countries’ interventions, to identify the influence of each study. The analysis was presented in a forest plot and performed in RevMan 5.4.1. software (The Cochrane Collaboration).

To assess the certainty of the evidence, we followed the guidelines of the Grading of Recommendations, Assessment, Development, and Evaluation (GRADE) working group. The GRADE system classifies the quality of evidence into 4 categories: high, moderate, low, and very low [27]. This evaluation was performed in GRADEpro GDT software [26].

#### Assessment of risk of bias in included studies

Two independent reviewers (SP-M and AG-R) assessed the risk of bias (RoB) in each study. In cases of disagreement, a consensus was reached through discussion among the 3 authors (SP-M, AG-R, and EK-H). The RoB tool and the RoB in Non-randomized Studies of Interventions tool were used following the *Cochrane Handbook for Systematic Reviews of Interventions* criteria [22]. Each domain was graded as a high, low, or unclear risk. We used the RobVis package [28] for the graphs and summary.

## Results

### Search results

An electronic and manual search was conducted in February 2024 and updated in January 2025, resulting in the identification of 11,170 titles and abstracts. We found a total of 2961 duplicate titles. In addition, 7978 titles were not eligible because they did not meet the specified inclusion criteria. Of the 231 studies selected for full-text review, 59 were excluded for being systematic reviews or international guidelines, 12 due to unavailability of the complete text, 2 because they were developed in a different setting from primary health care, 51 because they did not focus on any form of malnutrition in pregnant women, 6 for not addressing the interventions of interest, 52 because the study design did not include key elements for implementing the studied interventions, and 7 because they included women with a previous diagnosis of conditions such as gestational diabetes or anemia. Consequently, 51 articles were included (Figure 1). Table 2 [[29], [30], [31], [32], [33], [34], [35], [36], [37], [38], [39], [40], [41], [42], [43], [44], [45], [46], [47], [48], [49], [50], [51], [52], [53], [54], [55], [56], [57], [58], [59], [60], [61], [62], [63], [64], [65], [66], [67], [68]] summarizes the main characteristics of the identified studies, and Table 3 presents the impact of the interventions on malnutrition outcomes.

**TABLE 2:** Characteristics of the analyzed studies in a systematic review of effective implementation strategies for delivering nutritional interventions to prevent malnutrition during pregnancy.

Study ID	Country	Study design	Intervention type	What	Based on a model	Based on guideline	Resources or materials	Provider	Delivery mode	How	Where	When	Frequency	Dose	Duration	Tailored
Thaver et al. [29]	Pakistan	QEWCG	Supplementation; Healthy eating	Multiple micronutrient supplementation (UNIMMAP formula) and advice for consumption + counseling on healthy eating	No	Yes	Yes	CHW	Individual	Face-to-face	Antenatal clinics-home visits	1st trimester	Monthly	1 tablet/d	Until delivery	No
Schulze et al. [30]	Bangladesh	RCT	Supplementation	Micronutrient supplementation (UNIMMAP formula adapted)	No	Yes	Yes	CHW	Individual	Face-to-face	Home visits	1st trimester	Weekly	1 tablet/d	During pregnancy and until 12 wk postpartum	No
Roberfroid et al. [31]	Burkina Faso	RCT	Supplementation	Micronutrient supplementation (UNIMMAP formula)	No	Yes	Yes	CHW	Individual	Face-to-face	Health centers	1st trimester	Daily	1 tablet/d	During pregnancy and until 12 wk postpartum	Yes
Ramakrishnan et al. [32]	Vietnam	RCT	Supplementation	Micronutrient supplementation (differs UNIMMAP formula) and advice for consumption	No	No	No	CHW	Individual	Mix	Home visits	1st trimester	Biweekly	2 capsules/d	Until delivery	No
Persson et al. [33]	Bangladesh	RCT	Supplementation	Micronutrient supplementation (UNIMMAP formula)	No	Yes	Yes	Doctor, nurse, paramedic	Individual	Face-to-face	Health centers-Home visits	1st/2nd trimester	Monthly	1 capsule/d	Until delivery	No
Mei et al. [34]	China	RCT	Supplementation	Micronutrient supplementation (UNIMMAP formula)	No	Yes	No	Doctor	Individual	Face-to-face	Health centers-home visits	1st/2nd trimester	Monthly	1 capsule/d	Until delivery	No
Liu et al. [35]	China	RCT	Supplementation	Micronutrient supplementation (UNIMMAP formula) and advice for consumption	No	Yes	No	Doctor	Individual	Face-to-face	Community clinics-home visits	1st/2nd trimester	Monthly	1 capsule/d	Until delivery	No
Kang et al. 2017 [36]	China	Cohort	Supplementation	Micronutrient supplementation (differs UNIMMAP formula) and advice for consumption	No	No	No	Doctor, CHW	Individual	Face-to-face	Community clinics-home visits	1st/2nd trimester	Monthly	1 tablet/d	Until delivery	Yes
Christian et al. [37]	Nepal	RCT	Supplementation	Micronutrient supplementation (differs UNIMMAP formula) and advice for consumption	No	No	Yes	CHW	Individual	Face-to-face	Home visits	1st trimester	Biweekly	1 tablet/d	During pregnancy and until 12 wk postpartum	No
Brough et al. [38]	United Kingdom	RCT	Supplementation	Micronutrient supplementation (differs UNIMMAP formula) and advice for consumption	No	No	Yes	NR	Individual	Face-to-face	Community clinics	1st/2nd trimester	Routine appointments (3)	1 tablet/d	Until delivery	No
Asemi et al. [39]	Iran	RCT	Supplementation	Micronutrient supplementation (differs UNIMMAP formula)	No	No	Yes	NR	Individual	Mix	Antenatal clinics	2nd trimester	Monthly	1 tablet/d	Until delivery	No
Adu-Afarwuah et al. [40]	Ghana	RCT	Supplementation	Micronutrient supplementation (UNIMMAP formula adapted) and advice for consumption	No	Yes	Yes	Nurse, CHW	Individual	Face-to-face	Antenatal clinics	1st/2nd trimester	Fortnightly	1 capsule/d	During pregnancy and the first 6 mo postpartum	Yes
				Micronutrient supplementation (UNIMMAP formula adapted + energy and macronutrients) and advice for consumption									Fortnightly	1 sachet/d	During pregnancy and the first 6 mo postpartum	
Thomson et al. [41]	United States	RCT	Healthy eating, physical activity, weight monitoring	Counseling on healthy eating and the development of physical activity habits, as well as for weight monitoring + weight assessment	Yes	Yes	Yes	CHW	Individual	Face-to-face	Home visits	2nd trimester	Monthly	Counseling session last 90–120 min	Until delivery	Yes
Téoule et al. [42]	Germany	RCT	Physical activity	Counseling primarily focused on physical activity and healthy lifestyle	No	Yes	Yes	Doctors, medical assistants, and midwives	Individual	Mix	Antenatal clinics	1st trimester	Monthly (videocalls and routine appoints until 32 wk), biweekly (after 32 wk)	Videocalls last 10–60 min	2 wk postpartum	Yes
Kinnunen et al. [43]	Finland	RCT	Healthy eating, physical activity, weight monitoring	Counseling on healthy eating and physical activity, as well as weight monitoring	Yes	Yes	Yes	Doctor, nurse	Individual	Face-to-face	Health centers	1st trimester	Routine appointments (5)	Counseling session last 10–30 min	37 wk	Yes
deJersey et al. 2022 [69]	Australia	QEWCG	Healthy eating, physical activity, weight monitoring	Counseling on healthy eating and physical activity, as well as weight monitoring	Yes	Yes	Yes	Doctor, nutritionist, midwife	Individual	Face-to-face	Antenatal clinics	2nd trimester	Routine appointments (8)	Not mentioned	36 wk	Yes
Wakwoya et al. [44]	Ethiopia	RCT	Healthy eating	Intensive nutrition education and healthy eating counseling	Yes	Yes	Yes	Midwife	Individual	Mix	Health centers	1st/2nd trimester	Routine appointments (3) and 18 text messages (weekly)	Counseling session lasts for 30–45 min, and 18 text messages were delivered	3rd trimester	Yes
Wakwoya et al. [45]	Ethiopia	RCT	Healthy eating	Intensive nutrition education and healthy eating counseling	Yes	Yes	Yes	Midwife	Individual	Mix	Health centers	1st/2nd trimester	Routine appointments (3) and 18 text messages (weekly)	Counseling session lasts for 30 min, and 18 text messages were delivered	3rd trimester	Yes
Sisay and Tesfaye [46]	Ethiopia	RCT	Healthy eating	Nutrition education and healthy eating counseling	No	Yes	Yes	Nutritionist	Mix	Face-to-face	Health centers	2nd trimester	4 monthly sessions	Counseling session last 60 min	3rd trimester	Yes
Husaini et al. [47]	Indonesia	Cohort	Weight-gain monitoring	Weight assessment	No	No	Yes	CHW	Individual	Face-to-face	Health centers	1st/2nd trimester	Monthly	—	Until delivery	No
Huang et al. [48]	Australia	RCT	Healthy eating, physical activity, weight monitoring	Counseling on healthy eating and physical activity, as well as for weight monitoring + weight assessment	Yes	Yes	Yes	Nutritionist	Individual	Mix	Antenatal clinics	1st trimester	Weekly	Not mentioned	1st trimester -12 wk postpartum	Yes
Habtu et al. [49]	Rwanda	QEWCG	Healthy eating	Nutrition education and healthy eating counseling	No	Yes	No	CHW-nutritionist	Individual	Face-to-face	Health centers	1st trimester	Routine appointments (unmentioned frequency)	Counseling session last 30–45 min	3rd trimester	Yes
Garmendia et al. [50]	Chile	RCT	Healthy eating, physical activity	Counseling on healthy eating and physical activity	No	Yes	Yes	Midwife	Individual	Face-to-face	Health centers	1st trimester	Routine appointments (6)	Counseling session last 60 min	—	No
Di Carlo et al. [51]	Italy	RCT	Healthy eating; Weight-gain monitoring	Counseling on healthy eating + weight-gain monitoring	No	Yes	No	Nutritionist	Individual	Face-to-face	Antenatal clinics	1st trimester	Monthly	Not mentioned	37 wk	Yes
Abdel-Aziz et al. [52]	Egypt	RCT	Healthy eating, physical activity, weight monitoring	Counseling on healthy eating and physical activity, as well as for weight monitoring + weight assessment	No	Yes	Yes	Nutritionist	Individual	Mix	Antenatal clinics	1st trimester	Routine appointments (11–15) + 6 additional sessions	Counseling session last 20–30 min	Until delivery	Yes
Vítolo et al. [53]	Brazil	RCT	Healthy eating, weight-gain monitoring	Counseling on healthy eating + weight-gain monitoring	No	Yes	No	NR	Individual	Face-to-face	Health centers	1st/2nd trimester	3 sessions	Not mentioned	36 wk	Yes
Trotman et al. [54]	United States	Cohort	Healthy eating, physical activity, weight monitoring	Counseling on healthy eating and physical activity, as well as for weight monitoring + weight assessment	Yes	No	No	Doctor, midwife	Mix	Face-to-face	Antenatal clinics	1st/2nd trimester	Monthly, biweekly, weekly	Counseling session last 120 min	Until delivery	Yes
Tarqui-Mamani et al. [55]	Peru	QEWCG	Healthy eating	Counseling on healthy eating	No	Yes	Yes	NR	Individual	Virtual	Health centers	1st/2nd trimester	At least every 3 d	Not mentioned	3rd trimester	No
Tanner-Smith et al. 2014 [56]	United States	Cohort	Healthy eating, physical activity, weight monitoring	Counseling on healthy eating and physical activity, as well as for weight monitoring + weight assessment	Yes	Yes	Yes	Doctor, nurse, midwife	Mix	Face-to-face	Health centers	1st trimester	10 sessions	Counseling session last 90–120 min	Until delivery	Yes
Saidi et al. [57]	Canada	QEWCG	Physical activity, weight-gain monitoring	Counseling on healthy eating and physical activity, as well as for weight control + weight assessment	Yes	Yes	Yes	Doctor, nurse	Individual	Face-to-face	Antenatal clinics	1st trimester	Routine appointments (8)	Not mentioned	37 wk	No
Reyes et al. [58]	Mexico	Cohort	Healthy eating, weight-gain monitoring	Intensive nutrition education and healthy eating counseling + weight-gain monitoring	No	Yes	No	Doctor	Individual	Face-to-face	Antenatal clinics	1st trimester	Monthly, biweekly, weekly	Not mentioned	Until delivery	No
Magriples et al. [59]	United States	RCT	Healthy eating, physical activity, weight monitoring	Counseling on healthy eating and physical activity, as well as for weight monitoring + weight assessment	Yes	Yes	No	Doctor, midwife, assistant	Group	Face-to-face	Health centers	1st/2nd trimester	10 sessions	Counseling session last 120 min	6 mo pospartum	Yes
Kunath et al. [60]	Germany	RCT	Healthy eating, physical activity, weight monitoring	Counseling on healthy eating and physical activity, as well as for weight monitoring + weight assessment	No	Yes	Yes	Doctor, midwife, assistant	Individual	Face-to-face	Health centers	1st trimester	3 sessions	Counseling session last 30–45 min	6–8 wk postpartum	Yes
Krebs et al. [61]	Germany	RCT	Healthy eating, physical Activity	Counseling on healthy eating and physical activity	No	Yes	Yes	Doctor, midwife	Individual	Mix	Antenatal clinics	1st trimester	6 sessions	10 min per session	Until delivery	Yes
Gesell et al. [62]	United States	RCT	Healthy eating, physical activity	Counseling on healthy eating and physical activity	Yes	No	No	Health care providers	Group	Face-to-face	Community recreation	1st-3rd trimester	Weekly	90 min per session	Until delivery	Yes
deJersey et al. 2022	Australia	QEWCG	Healthy eating, physical activity, weight monitoring	Counseling on healthy eating and physical activity, as well as for weight monitoring + weight assessment	Yes	Yes	Yes	Nutritionist	Individual	Virtual	Antenatal clinics	1st/2nd trimester	10 phone calls	Phone calls last 60 min	36 wk	Yes
Daley et al. [63]	England	RCT	Weight-gain monitoring	Weight assessment according to BMI	Yes	Yes	Yes	Midwife	Individual	Face-to-face	Antenatal clinics	1st/2nd trimester	Routine appointments (8)	Not mentioned	38 wk	No
Coughlin et al. [64]	United States	RCT	Healthy eating, physical activity, weight monitoring	Counseling on healthy eating and physical activity, as well as for weight monitoring + weight assessment	Yes	Yes	Yes	Nutritionist	Individual	Virtual	Antenatal clinics	1st/2nd trimester	Weekly and biweekly	Counseling session last 20–30 min	12 wk postpartum	Yes
Brumley et al. [65]	United States	Case control	Healthy eating, physical activity, weight monitoring	Counseling on healthy eating and physical activity, as well as for weight monitoring	No	Yes	Yes	Midwife, physical therapist	Group	Face-to-face	Antenatal clinics	1st/2nd trimester	Monthly	Not mentioned	36–40 wk	Yes
Atkinson et al. [66]	Canada	RCT	Healthy eating, physical activity, weight monitoring	Counseling on healthy eating and physical activity, as well as for weight monitoring	No	Yes	Yes	Doctor, nutritionist, midwife, psychologist	Individual	Face-to-face	Health centers	1st/2nd trimester	Weekly and biweekly	Not mentioned	Until end of pregnancy	Yes
Asci et al. [67]	Turkey	RCT	Healthy eating, physical activity, weight monitoring	Counseling on healthy eating and physical activity, as well as for weight monitoring + weight assessment	Yes	Yes	Yes	Midwife	Individual	Mix	Health centers	1st trimester	Not mentioned	Counseling session lasts 60 min	37 wk	Yes
Asbee et al. 2009 [68]	United States	RCT	Healthy eating, physical activity, weight monitoring	Counseling on healthy eating and physical activity, as well as for weight monitoring + weight assessment	No	Yes	No	Doctor, nutritionist	Individual	Face-to-face	Antenatal clinics	1st/2nd trimester	Routine appointments	Not mentioned	Until end of pregnancy	Yes
Ramakrishnan 2004 [70]	Mexico	RCT	Supplementation	Micronutrient supplementation (including Fe) vs. Iron only	No	Yes	No	NR	Individual	Face-to-face	Antenatal clinics, home visits	1st trimester	Weekly	1 tablet/d	Until delivery	No
Vaidya 2008 [71]	Nepal	RCT	Supplementation	Micronutrient supplementation (15 vitamins/minerals) vs. iron + folic acid	No	Yes	No	NR	Individual	Face-to-face	Antenatal clinics	2nd trimester	Monthly	1 tablet/d	Until delivery	No
SUMMIT Study Group 2008 [72]	Indonesia	RCT	Supplementation	Micronutrient supplementation (UNIMMAP formula vs. IFA) + advice for consumption	No	Yes	Yes	Midwife	Individual	Face-to-face	Community health clinics	1st/2nd trimester	Monthly	1 capsule/d	Until 90 d postpartum	Si
Tofail 2008 [73]	Bangladesh	RCT	Supplementation	Micronutrient supplementation	No	Yes	No	CHW	Mix	Face-to-face	Community nutrition centers, home visits	1st/2nd trimester	Weekly	1 capsule/d	Until delivery	No
Sunawang 2009 [74]	Indonesia	RCT	Supplementation	Micronutrient supplementation (UNIMMAP formula vs. IFA)	No	Yes	No	NR	Individual	Face-to-face	Community health centers	1st trimester	Weekly	1 tablet/d	Until 30 d postpartum	No
West 2014 [75]	Bangladesh	RCT	Supplementation	Micronutrient supplementation (UNIMMAP formula vs. IFA)	No	Yes	No	NR	Individual	Face-to-face	Home visits	1st trimester	weekly	1 tablet/d	During pregnancy and until 12 wk postpartum	No
Moore 2009 [76]	Gambia	RCT	Supplementation	Micronutrient supplementation vs. Fe + FA and vs. protein-energy supplementation	No	Yes	No	CHW	Individual	Face-to-face	Antenatal clinics, home visits	1st trimester	Monthly	2 tablets/d	Until delivery	No
Zeng 2008 [77]	China	RCT	Supplementation	Micronutrient supplementation (UNIMMAP formula vs. IFA and vs. FA only) + advice for consumption	No	Yes	No	Doctor	Individual	Face-to-face	Antenatal clinics, home visits	1st trimester	Monthly	1 capsule/d	Until delivery	No
Bhutta 2009 [78]	Pakistan	RCT	Supplementation	Micronutrient supplementation (UNIMMAP formula vs. IFA) + counseling on maternal nutrition and antenatal care	No	Yes	Yes	CHW/social scientist with training in nutrition	Individual	Face-to-face	Home visits	1st trimester	Biweekly	1 tablet/d	Until delivery	No

Abbreviations: CHW, community health worker; NR, not reported; QEWCG, Quasi-Experimental Study with Control Group; RCT, randomized controlled trial; UNIMMAP, United Nations International Multiple Micronutrient Antenatal Preparation.

**TABLE 3:** Results associated with malnutrition indicators in pregnant women by type of intervention.

Study	Intervention type	Indicator		*P* value
Schulze et al. [30]	Supplementation	Concentration of Hb concentration (g/L) in late pregnancy at 32 wk (mean difference, 95% CI)	−0.5 (−1.4, 0.5)	0.341
		Hb percentage in plasma indicators in late pregnancy (difference)	−4%	0.365
		Concentration of Ferritin concentration (μg/L) in late pregnancy at 32 wk (mean difference, 95% CI)	−3.8 (−6.4, −1.3)	0.003
		Ferritin percentage in plasma indicators in late pregnancy (difference)	−9%	0.008
		Concentration of TfR (mg/L) in late pregnancy at 32 wk (mean difference, 95% CI)	0.1 (−0.3, 0.5)	496
		Percentage of TfR in plasma indicators in late pregnancy at 32 wk (difference)	5.90%	0.364
Roberfroid et al. [31]	Supplementation	Micronutrient supplement (UNIMMAP or IFA) relation to anemia at the second measurement, OR (95% CI)	0.84 (0.63, 1.13)	>0.05
		Anemia at inclusion (yes or no), relation to anemia at the second measurement, OR (95% CI)	2.38 (1.74, 3.23)	0.0001
		Hb concentration at baseline (g/dL), relation to recovery from baseline anemia OR (95% CI)	1.38(1.05, 1.80)	<0.05
		Hb change (g/dL) per tablet in women with baseline anemia	0.006 ± 0.001	0.001
		Hb change (g/dL) per tablet in women without baseline anemia	−0.003 ± 0.001	0.002
Ramakrishnan et al. [32]	Supplementation	Changes in mean Hb (mean ± SD) from baseline to late pregnancy (MMS vs. IFA)	–1.39 ± 1.65	≥0.05
		Changes in mean Hb (mean ± SD) from baseline to late pregnancy (FA vs. MMS)	−1.59 ± 1.63	≥0.05
Persson et al. [33]	Supplementation	Maternal Hb concentration at 30 wk of gestation (g/L) 95% CI (60-mg iron + 400-μg FA vs. MMS)	114.9 vs. 114.9 difference −0.4 (−1.5, 0.6)	0.97
		Difference in mean maternal Hb concentration (g/L) 95% CI early vs. usual invitation to food supplementation groups	−0.9 (−1.7, 0.1)	0.04
Mei et al. [34]	Supplementation	Abnormal Hb concentration (<110 g/L), FA vs. MMS % (95% CI)	5.3 (2.7, 7.9) vs. 3.3 (1.2, 5.4)	<0.05
		Abnormal Hb concentration (<110 g/L), iron-FA vs. MMS % (95% CIs)	7.2 (4.2, 10.2) vs. 3.3 (1.2, 5.4)	<0.05
		Abnormal SF concentration (<12 mg/L), FA vs. MMS % (95% CIs)	59.6 (53.8, 65.3) vs. 42.7 (36.8, 48.6)	<0.05
		Geometric means (95% CIs) for SF mg/L (baseline vs. follow-up) in MMS group	55.6 (50.7, 61.0) vs. 15.0 (13.7, 16.5)	<0.001
		Geometric means (95% CIs) for STfR, mg/L (baseline vs. follow-up) in MMS group	4.06 (3.86, 4.27) vs. 4.34 (3.84, 4.89)	0.309
		Arithmetic means (95% CIs) for Hb (g/L) (baseline vs. follow-up) in MMS group	121.5 (120.6, 122.5) vs. 124.4 (123.2, 125.6)	<0.001
		Geometric means (95% CIs) for SF mg/L, FA vs. MMS	11.3 (10.4, 12.1) vs. 15.0 (13.7, 16.5)	<0.05
		Geometric means (95% CIs) for STfR (mg/L), FA vs. MMS	4.79 (4.29, 5.34) vs. 4.34 (3.84, 4.89)	<0.05
		Geometric means (95% CIs) for STfR (mg/L), IFA vs. MMS	3.99 (3.52, 4.53) vs. 4.79 (4.29, 5.34)	<0.05
Liu et al. [35]	Supplementation	Hb concentration, g/dL, mean difference (95% CI) MMS vs. FA	0.06 (0.03, 0.09)	0.001
		Hb concentration, g/dL, mean difference (95% CI) MMS vs. IFA	0.02 (−0.02, 0.05)	0.34
		Anemia, RR (95% CI) Hb concentration <11.0 g/dL MMS vs. FA	0.71 (0.62, 0.82)	0.001
		Anemia, RR (95% CI) Hb concentration <11.0 g/dL MMS vs. IFA	0.99 (0.85, 1.15)	0.89
Kang et al. [36]	Supplementation	Anemia MMS vs. FA at 32.2 wk of gestation (adjusted OR 95% CI)	0.63(0.45, 0.8)	0.007
		Hb concentration (g/L) MMS vs. FA at 32.2 wk of gestation, (mean difference)	5.18; (2.98, 7.37)	<0.001
Christian et al. [37]	Supplementation	Hb (g/L) Mean (95% CI), relative difference, MMS vs. control	9.4 (4.7, 14.1)	<0.001
		Severe anemia (Hb<70 g/L) (95% CI), prevalence ratio, MMS vs. control	0.48 (0.18, 1.31)	0.15
		Mild-to-moderate anemia (Hb 70–109 g/L) (95% CI), prevalence ratio, MMS vs. control	0.63 (0.48, 0.83)	<0.001
		Any anemia (Hb <110 g/L) (95% CI), prevalence ratio, MMS vs. control	0.64 (0.48, 0.84)	<0.002
		Iron deficiency anemia (Hb <110 g/L and serum ferritin <12 μg/L) (95% CI), prevalence ratio, MMS vs. control	0.40 (0.27, 0.61)	<0.001
Brough et al. [38]	Supplementation	Hb (g/l) at 34 wk of gestation vs. placebo. Mean (SD)	113 (12) vs. 109 (10)	0.003
Asemi et al. [39]	Supplementation	Mean weight (kg) at delivery. Multivitamin vs. multivitamin–mineral supplement	74.0 ± 6.4 vs. 76.8 ± 5.8	0.02
		Mean BMI at delivery. Multivitamin vs. multivitamin–mineral supplement	29.5 ± 1.4 29.2 ± 1.5	0.22
Adu-Afarwuah et al. [40]	Supplementation	MMNs and IFA. Inadequate GWG. RR (95% CI)	1.1 (0.9, 1.2)	0.5
		MMNs and IFA. Adequate GWG. RR (95% CI)	0.9 (0.7, 1.3)	0.9
		MMNs and IFA. Excessive GWG. RR (95% CI)	0.8 (0.4, 1.3)	0.48
		LNSs and MMNs. Inadequate GWG. RR (95% CI)	0.9 (0.7, 1.0)	0.023
		LNSs and MMNs. Adequate GWG. RR (95% CI)	1.3 (1.0, 1.7)	0.11
		LNSs and MMNs. Excessive GWG. RR (95% CI)	1.3 (0.8, 2.3)	0.41
Ramakrishnan 2004	Supplementation	Mean Hb concentrations (g/L) at 32 wk of gestation (95% CI) IG vs. CG	104.2 (102.5, 106.0) vs. 108.1 (106.4, 109.8)	<0.01
		Mean Hb concentrations at 1 mo postpartum (95% CI) IG vs. CG	117.5 (115.3, 119.7) vs. 119.7 (117.6, 121.9)	>0.01
		% of anemia at 32 wk of gestation (Hb <105 g/L) IG vs. CG	47.9 vs. 42.6	>0.05
		% of anemia at 1 mo postpartum (Hb <120 g/L) IG vs. CG	47.2 vs. 49.3	>0.05
		% of Iron deficiency (SF <12.0 μg/L) at 32 wk of gestation IG vs. CG	90.9 vs. 92.6	>0.05
		% of Iron deficiency (SF <12.0 μg/L) at 1 mo postpartum IG vs. CG	45.1 vs. 47.3	>0.05
		% of Iron deficiency anemia at 32 wk of gestation (Hb <105 g/L and SF <12.0 μg/L) IG vs. CG	45.8 vs. 40.5	>0.05
		% of Iron deficiency anemia at 1month postpartum (Hb <120 g/L and SF <12.0 μg/L) IG vs. CG	29.6 vs. 31.8	>0.05
Vaidya 2008	Supplementation	Mean Hb (mmol/L) (SD) (IG vs. CG)	6.98 (0.79) vs. 6.96 (0.83)	Not showed
SUMMIT study group	Supplementation	Hb concentration (%) <110 g/L at first trimester. IFA vs. MMN	27% vs. 29%	Not showed
		Hb concentration (%) <110 g/L at second trimester. IFA vs. MMN	56% vs. 56%	Not showed
		Hb concentration (%) <110 g/L at third trimester. IFA vs. MMN	59% vs. 61%	Not showed
Tofail 2008^1^	Supplementation	Hb mean difference (95% CI)	0.10 (–1.00, 1.20)	Not showed
Sunawang 2009^1^	Supplementation	Hb mean difference (95% CI)	–2.00 (–3.84, –0.16)	Not showed
Moore 2019^1^	Supplementation	Hb mean difference (95% CI)	1.30 (1.06, 1.54)	Not showed
West 2014	Supplementation	Mean Hb g/L mean (SD) at 3rd trimester. IFA vs. MMN	111.1 (10.3) vs. 110.7 (10.4)	>0.05
		Mean Hb g/L mean (SD) 3 mo postpartum. IFA vs. MMN	120.7 (9.8) vs. 120.9 (9.7)	>0.05
		Prevalence of anemia (<110 g/L) at 3rd trimester. IFA vs. MMN	42.4% vs. 42.8%	>0.05
		Prevalence of anemia (<110 g/L) 3 mo postpartum. IFA vs. MMN	12.0% vs. 11.5%	>0.05
Zeng 2008	Supplementation	Adjusted difference in Hb (g/L). MMN vs. FA	6.9 (4.1, 9.6)	<0.001
		Adjusted RR for anemia (Hb <110 g/L). MMN vs. FA	0.72 (0.59, 0.88)	0.001
Buttha 2009	Supplementation	Maternal monthly weight gain (kg). Mean ± SD. IFA vs. MMN	1.24 ± 0.8 vs. 1.38 ± 1.0	<0.001
		Maternal monthly gain in MUAC (cm). Mean ± SD. IFA vs. MMN	0.18 ± 0.6 vs. 0.19 ± 0.6	0.70
		Hb (g/dL) postsupplementation. Mean ± SD. IFA vs. MMN	10.9 ± 1.6 vs. 10.9 ± 1.6	0.27
		Ferritin (ng/dL) Mean ± SD. IFA vs. MMN	48.9 ± 39.1 vs. 40.0 ± 37.2	<0.001
Thomson et al. [41]	HE, PA, and weight gain	Weight below IOM recommendations (PAT vs. PATE)	20.9 % vs. 28.8%	0.192
		Weight within IOM recommendations (PAT vs. PATE)	25.6% vs. 10.3%	
Kinnunen et al. [43]	HE, PA, and weight-gain monitoring	GWG below recommendations IG vs. CG, *n* (%)	16 (33) vs. 15 (27)	0.053
		GWG within recommendations IG vs. CG, *n* (%)	10 (21) vs. 24 (43)	
		GWG, exceeded recommendations IG vs. CG, *n* (%)	22 (46) vs. 17 (30)	
		Adjusted OR (95% CI) for exceeding recommendations. Ref CG	1.82 (0.65, 5.14)	0.26
DeJersey 2022	HE, PA, and weight-gain monitoring	Exceeded IOM guidelines for GWG. LWdP intervention group and historical comparison group	86 (70%) vs. 30 (73%)	0.69
		Total GWG until 36 wk (kg). LWdP intervention group and historical comparison group	14.7 (7.6) vs. 13.5 (8.8)	0.41
Huang et al. [48]	HE, PA, and weight-gain monitoring	Adherence to IOM guidelines for GWG—inadequate weight gain, CG vs. IG. n (%)	6 (31.58%) vs. 5 (21.74%)	0.47
		Adherence to IOM guidelines for GWG—recommended weight gain. CG vs. IG. *n* (%)	2 (10.53%) vs. 5 (21.74%)	0.428
		Adherence to IOM guidelines for GWG—excessive weight gain. *n* (%) CG vs. IG	11 (57.89%) vs. 13 (56.52%)	0.929
Abdel-Aziz et al. [52]	HE, PA, and weight-gain monitoring	GWG within the IOM recommendations (IG vs. CG) between the 25th and 35th wk of gestation (%)	42.7% vs. 13.9%	<0.001
		GWG—below recommendations (% IG vs. CG)	6.6 vs. 11.1	<0.001
		GWG—within recommendations (% IG vs. CG)	42.7 vs. 13.9	
		GWG—above recommendations (% IG vs. CG)	50.7 vs. 75	
		Anemia (Hb concentration <11 g/dL) % IG vs. CG	12 vs. 27.8	<0.016
Trotman et al. [54]	HE, PA, and weight-gain monitoring	Met IOM GWG guidelines (%) CPPC vs. SPPC	62 vs. 38	0.02
		Met IOM GWG guidelines (%) MPPC vs. SPPC	28 vs. 38	1
Tanner-Smith et al. [56]	HE, PA, and weight-gain monitoring	High GWG vs. Healthy GWG CP prenatal care (vs. TC) (b, SE, CI95%, RRR)	−0.99; 0.47 (−1.92, −0.06); 0.37	<0.05
		High GWG vs. healthy GWG CP prenatal care (vs. TC) (overweight vs. normal weight) (b, SE, CI95%, RRR)	1.21; 0.42 (0.39, 2.02): 3.34	<0.05
		High GWG vs. healthy GWG CP prenatal care (vs. TC) (obese vs. normal weight) (*b*, SE, 95% CI, RRR)	1.44; 0.47 (0.51, 2.37): 4.22	<0.05
Magriples et al. [59]	HE, PA, and weight-gain monitoring	Half of the participants exceeded GWG amounts based on IOM guidelines		—
Kunath et al. [60]	HE, PA, and weight-gain monitoring	Weight above IOM recommendations. OR (95% CI)	0.95 (0.66, 1.38)	0.789
		% weight above the IOM recommendations. IG vs. CG	45.1 vs. 45.7	>0.05
DeJersey 2022	HE, PA, and weight-gain monitoring	Total GWG in normal weight women preimplementation and postimplementation (kg)	(14.2 ± 5.3 vs. 13.3 ± 4.7)	0.04
		Proportion of women with excess GWG between preimplementation and postimplementation in normal-weight women	(31% vs. 24%)	0.035
		Reduction in excess GWG between preimplementation and postimplementation in normal-weight women. Adjusted OR 95% CI	0.53 (95% CI: 0.29, 0.96)	0.005
Coughlin et al. [64]	HE, PA, and weight-gain monitoring	Participants who met IOM weight-gain guidelines at 37 wk of pregnancy, H42/H4U group vs. the health education comparison group (%)	77% vs. 54%	0.41
Brumley et al. [65]	HE, PA, and weight-gain monitoring	Excessive weight gain, lbs [mean (SD)]. IG vs. CG	9.64 (5.8) vs. 10.61 (8.66)	0.24
Atkinson et al. [66]	HE, PA, and weight-gain monitoring	Women who achieved GWG within the IOM recommendations. IG vs. CG	1.51; 95% CI (0.81, 2.80)	>0.05
Asci et al. [67]	HE, PA, and weight-gain monitoring	GWG within IOM recommendations between the groups (OR)	0.379 (0.141, 1.021)	0.05
Asbee et al. [68]	HE, PA, and weight-gain monitoring	Adherence to the IOM guidelines between groups (%). IG vs. CG	61.4 vs. 48.8	0.21
		Adherence to the IOM guidelines (OR 95% CI ref IG)	0.426 (0.148, 1.231)	0.115
Thaver et al. [29]	Supplementation + HE	Hb concentration (mg/dL) (mean) at 3rd visit: IG vs. CG	11.1 ± 1.4 vs. 10.2±1.6	0.00
		Hb concentration (mg/dL) (gain% IG vs. CG)	11.9 vs. 2.9	<0.001
		kg/m^2^ (mean) 3rd visit IG vs. CG	15.8 ± 2.2 vs. 15.4 ± 2.1	0
Saidi and Tesfaye [57]	PA + weight-gain monitoring	Excessive GWG: OR (95% CI). Reference: adequate GWG	0.71 (0.49, 0.99)	Not showed
		Adequate weight gain by BMI BMI < 18.5: OR (95% CI)	0.17 (0.02, 1.42)	Not showed
		Adequate weight gain by BMI BMI 18.5–24.9 kg/m^2^: OR (95% CI)	1.35 (0.83, 2.22)	Not showed
		Adequate weight gain by BMI BMI 25.0–29.9 kg/m^2^: OR (95% CI)	0.84 (0.30, 2.34)	Not showed
		Adequate weight gain by BMI BMI ≥30 kg/m^2^: OR (95% CI)	3.37 (1.31 8.67)	Not showed
Di Carlo et al. [51]	HE + weight-gain monitoring	Women with a healthy pregnancy weight gain (equal or lower than 12 kg) IG vs. CG. % (*n*)	95.1 % (58) vs. 41% (25)	<0.001
		Women with a healthy pregnancy weight gain (equal or lower than 12 kg) IG vs. CG. RR (95 % CI)	2.4 (1.9, 2.5)	<0.001
Vítolo et al. [53]	HE + weight-gain monitoring	Weekly maternal weight gain. Woman with normal weight—mean ± SD, IG vs. CG (g)	460.7 ± 135.2 vs. 492.2 ± 222.1	0.2
		Weekly maternal weight gain. Woman with obesity- mean ± SD, IG vs. CG (g)	342.2 ± 143.6 vs. 420.2 ± 185.4	0.01
		Prevalence of overweight in pregnant women, who gained >10 kg—RR (95 % CI)	0.4 (0.19, 0.96)	Not showed
Reyes et al. [58]	HE + weight-gain monitoring	GWG (kg) mean ± SD Women with obesity (OB) vs. normal-weight women (NW)	6.1 ± 4.4 vs. 10.3 ± 5.4	<0.001
		GWG (kg) mean ± SD Overweight women (OW) vs. NW women	9.5 ± 5.1 vs. 10.3 ± 5.4	>0.05
		% Weight gain below recommended by IOM NW vs. OW	31.3 vs. 14	0.0001
		% Weight gain below recommended by IOM NW vs. OB	31.3 vs. 20.7	0.02
		% Weight gain below recommended by IOM OW vs. OB	14 vs. 20.7	>0.05
		% Weight gain above recommended by IOM NW vs. OW	17 vs. 35.7	0.0001
		% Weight gain above recommended by IOM NW vs. OB	17 vs. 22.4	< 0.01
		% Weight gain above recommended by IOM OW vs. OB	35.7 vs. 22.4	>0.05
Garmendia et al. [50]	HE + PA	GWG within IOM recommendations Treatment effect [OR (95% CI)]	0.94 (0.81, 1.09)	Not showed
		GWG within IOM recommendations, pregestational normal weight. Treatment effect [OR (95% CI)]	0.73 (0.54, 0.97)	Not showed
		GWG within IOM recommendations, pregestational overweight. Treatment effect [OR (95% CI)]	1.21 (0.93, 1.57)	Not showed
		GWG within IOM recommendations, pregestational obesity. Treatment effect [OR (95% CI)]	1.08 (0.78, 1.50)	Not showed
		GWG >IOM recommendations Treatment effect [OR (95% CI)]	0.96 (0.77, 1.19)	Not showed
		GWG >IOM recommendations, pregestational normal weight. Treatment effect [OR (95% CI)]	1.32 (0.96, 1.82)	Not showed
		GWG >IOM recommendations, pregestational overweight. Treatment effect [OR (95% CI)]	0.74 (0.49, 1.13)	Not showed
		GWG >IOM recommendations, pregestational obesity. Treatment effect [OR (95% CI)]	0.75 (0.52, 1.08)	Not showed
		GWG <IOM recommendations, treatment effect [OR (95% CI)]	1.21 (1.01, 1.44)	Not showed
		GWG <IOM recommendations, pregestational normal weight. Treatment effect [OR (95% CI)]	1.41 (1.04, 1.91)	Not showed
		GWG <IOM recommendations, pregestational overweight. Treatment effect [OR (95% CI)]	0.93 (0.66, 1.32)	Not showed
		GWG <IOM recommendations, pregestational obesity. Treatment effect [OR (95% CI)]	1.18 (0.78, 1.76)	Not showed
Krebs et al. [61]	HE + PA	GWG reduction in the intervention group (kg, 95% CI)	1, –1.56, –0.38	<0.001
		Women who had experienced excessive GWG, CG vs. IG adjusted OR (95% CI)	0.76 (0.60, 0.96)	0.024
		Women who had experienced excessive GWG by BMI < 18.5 kg/m^2^ CG vs. IG	1.30 (0.41, 4.08)	0.605
		Women who had experienced excessive GWG by BMI 18.5–24.9 kg/m^2^ CG vs. IG	0.71 (0.52, 0.97)	0.031
		Women who had experienced excessive GWG by BMI 25.0–29.9 kg/m^2^ CG vs. IG	0.84 (0.45, 1.54)	0.566
		Women who had experienced excessive GWG by BMI ≥30.0 kg/m^2^ CG vs. IG	0.87 (0.51, 1.49)	0.658
Gesell et al. [62]	HE + PA	Normal weight *n* (%) within IOM recommendations. CG vs. IG	3 (17.6) vs. 6 (40.0)	0.36
		Normal weight *n* (%) over IOM recommendations. CG vs. IG	8 (47.1) vs. 1 (6.7)	
		Overweight *n* (%) under IOM recommendations. CG vs. IG	6 (40.0) vs. 3 (21.4)	0.227
		Overweight *n* (%) within IOM recommendations. CG vs. IG	3 (20.0) vs. 7 (50.0)	
		Overweight *n* (%) over IOM recommendations. CG vs. IG	6 (40.0) vs. 4 (28.6)	
		Obese *n* (%) under IOM recommendations. CG vs. IG	1 (9.1) vs. 4 (26.7)	0.434
		Women with obesity *n* (%) within IOM recommendations. CG vs. IG	5 (45.5) vs. 4 (26.7)	
		Women with obesity *n* (%) over IOM recommendations. CG vs. IG	5 (45.5) vs. 7 (46.7)	
		All n (%) under IOM recommendations. CG vs. IG	13 (30.2) vs. 15 (34.1)	0.223
		All *n* (%) within IOM recommendations. CG vs. IG	11 (25.6) vs. 17 (38.6)	
		All *n* (%) over IOM recommendations. CG vs. IG	19 (44.2) vs. 12 (27.3)	
		Mean GWG (lbs) CG vs. IG. Mean (SD), *t*-test	22.41 (15.56) vs. 19.50 (12.27) *t*-test (*t* = –0.894)	0.374
Wakwoya et al. [44]	HE	Mean Hb difference of the differences between intervention and control measurement	0.45(1.57) vs. –0.01(1.81)	0.04
		Hb concentration estimate (SE) 95% CI	*b* = 0.50 (0.08) 0.33, 0.67	<0.01
Wakwoya et al. [45]	HE	MUAC (cm): IG vs. CG. Mean (SD)	23.26 (1.63) vs. 23.10 (1.67)	0.06
		Difference of the differences between the intervention and control groups MUAC (cm)	0.49 (1.23)	<0.01
		Proportion of undernutrition (%) IG vs. CG	25 vs. 36	<0.023
		Improved nutrition status at the end of the study trial estimate (SE) 95% CI	*b* = 0.47 (0.08) 0.33, 0.67	<0.01
Sisay and Tesfaye [46]	HE	Adjusted mean difference to controls Differences between baseline and end-line measurements (weight gain)	10.08 (9.55, 10.60)	0.001
		Adjusted mean difference to controls Differences between baseline and end-line measurements (MUAC)	1.27 (1.12, 1.42)	0
		Weight gain kg IG vs. CG	65.03 vs. 56.69 kg,	0.001
Habtu et al. [49]	HE	Anemia status: Anemic (Hb <11 g/dL) IG vs. CG (%)	10.5 (58) vs. 23.7 (129)	<0.001
		Normal (Hb ≥11 g/dL) IG vs. CG (%)	89.5 (494) vs. 76.3 (416)	
		Anemia status: Hb concentration, mean (SD) (IG vs. CG)	12.65 (1.24) vs. 12.10 (1.48)	<0.001
		Severity of anemia: prevalence, mild (Hb = 9–10.9 g/dL) (%) IG vs. CG	98.3 (57) vs. 86.8 (112)	0.014
		Severity of anemia: moderate (Hb = 7–8.9 g/dL) (%) IG vs. CG	1.7 (1) vs. 13.2 (17)	
		Acute wasting status: wasting (MUAC <23 cm) % IG vs. CG	3.4 (19) vs. 14.5 (79)	<0.001
		Acute wasting status: normal (MUAC ≥23 cm) % IG vs. CG	96.6 (533) vs. 85.5 (466)	
		Acute wasting status: MUAC, mean (SD)	26.06 (2.46) vs. 24.87 (2.45)	<0.001
		Underweight status in the first trimester: underweight (BMI <18.5 kg/m^2^) (%) IG vs. CG	1.8 (7) vs. 5.5 (25)	0.01
		Underweight status in the first trimester	76.2 (297) vs. 76.4 (349)	
		Underweight status in the first trimester: women with overweight/women with obesity (BMI >25.0 kg/m^2^) (%) IG vs. CG	22.1 (86) vs. 18.2 (83)	
		Underweight status in the first trimester: BMI, mean (SD) IG vs. CG	23.46 (2.97) vs. 22.73 (2.78)	<0.001
		Overall maternal undernutrition: undernourished (%) IG vs. CG	4.7 (26) vs. 18.2 (99)	<0.001
		Overall maternal undernutrition: normal (%) IG vs. CG	95.3 (526) vs. 81.8 (446)	
		Effect of the intervention on maternal undernutrition (woman who had low MUAC (<23 cm) during delivery or low BMI (<18.5 kg/m^2^) in the first trimester or both) Adjusted OR (95% CI)	0.23 (0.15, 0.36)	<0.001
Tarqui-Mamani et al. [55]	HE	Prevalence of anemia (%) CG vs. IG	5.1 vs. 13.8	>0.05
		Excessive weight gain % IG vs. CG	1.7 vs. 5.1	>0.05
Husaini et al. [47]	Weight-gain monitoring	32% Women improve their weight status from below the curve in the first trimester to above the curve in the second and third trimesters		—
Daley et al. [63]	Weight-gain monitoring	Proportion of women in the groups who gained weight within the IOM guidelines Adjusted OR (95% CI)	(OR: 0.92 95% CI: 0.63, 1.32)	0.63
		Proportion of women in the groups who gained weight less than the minimum IOM guidelines Adjusted OR (95% CI)	(OR: 1.26 95% CI: 0.86, 1.83)	0.24
		Adjusted mean difference in the change in weight (kg) during pregnancy Adjusted mean at 38 wk (95% CI)	–0.42 kg 95% CI: –1.49, 0.64	0.43
Téoule et al. [42]	PA	Below IOM GWG guidelines, *n* (%) control vs. intervention	8 (19) vs. 10 (22)	0.76
		Within IOM GWG guidelines, *n* (%) control vs. intervention	11 (26) vs. 21(47)	0.048
		Above IOM GWG guidelines, *n* (%) control vs. intervention	23 (55) vs. 14(31)	0.026

Abbreviations: AGP, α1-acid glycoprotein; CI, confidence interval; CG, control group; CP, centering pregnancy model; CPPC, centering pregnancy prenatal care; FA, folic acid; Fe, iron; GD, gestational diabetes; GWG, gestational weight gain; H42/H4U, remotely delivered health coaching program; Hb, hemoglobin; HE, healthy eating counseling; IFA, iron–folic acid; IG, intervention group; IOM, Institute of Medicine; LNS, lipid-based nutrient supplements; LWdP, living well during pregnancy program; MM, multiple micronutrient; MMS, multiple micronutrient supplement; MPPC, multiprovider prenatal care; MUAC, mid-upper arm circumference; NW, normal weight; OB, woman with obesity; OR, odds ratio; OW, overweight; PA, physical activity; PAT, parents as teachers group; PATE, nutrition and physical activity group; RR, relative risk; SF, serum ferritin; SPPC, single-provider prenatal care; SQ-LNSs, small-quantity lipid-based nutrient supplements; STfR, serum transferrin receptor; TC, traditional care; TfR, transferrin receptor; UNIMMAP, United Nations International Multiple Micronutrient Antenatal Preparation multiple micronutrient supplements; Zn, zinc.

1 Gomes et al. [79].

Of all included studies, 41.2% were conducted in high-income countries [38,[41], [42], [43],^48^,^50^,^51^,^54^,^80^,^69^, ^23^[38,41–43,48,50,51,54,70,71], 23.5% in upper-middle-income [[34], [35], [36],^47^,^53^,^55^,^58^,^68^, ^23^[34–36,47,53,55,58,68], 23.5% in lower-middle-income [[29], [30], [31], [32], [33],^37^,^39^,^40^,^44^,^52^,^67^], and 11.8% in low-income countries [45,46,49,81,82], according to the World Bank income classification. Although the studies analyzed represent all global regions, most originated from Asia (35.3%), particularly in Pakistan [32,52], Bangladesh [31,37,40,44], Nepal [33,83], Iran [30], Turkey [68], China [34,36,47], Vietnam [39], and Indonesia [35]. They were followed by studies from the Americas (19.6%), specifically Canada [42,62] and the United States [41,43,65,66,69]; Africa (15.7%) with studies from Ethiopia [53,81,82], Burkina Faso [49], Egypt [67], Gambia [46], Ghana [29], and Rwanda [45]; Europe (13.7%), with studies carried out in Germany [57,59,64], Finland [57], Italy [80], and the United Kingdom [38]; Latin America and the Caribbean (9.8%) with studies from Mexico, [53,84], Peru [55], Brazil [58], and Chile [51] and finally in Oceania (5.9%) with studies from Australia [50,56].

### Results per type of intervention

#### Micronutrient supplementation

Of the total number of studies, 20 were related to the delivery of micronutrient supplements, and 1 included 2 different types [Micronutrient capsules and Small-Quantity Lipid-Based Nutrient Supplements (SQ-LNS)] [29]. Regarding the study design, 19 were RCTs [38,36,47,29,30,33,37,39,40,31,44,46,49,83,85,84,[86], [87], [88]], and 1 was a cohort study [34]. About half of the studies in this category were from lower-middle-income countries (50%) [29,30,33,37,39,40,31,44,83,86].

The identified interventions had different dosage forms: 1 tablet/d (*n* = 11) [38,34,30,33,40,52,44,49,83,84,87, 2[38,34,30,33,40,52,44,49,74,75,78], 2 tablets/d (*n* = 1) [46, 2[46], 2 capsules/d (*n* = 1) [39, 1[39], 1 capsule/d (*n* = 7) [36,47,29,37,31,85,88], or 1 SQ-LNS sachetd (*n* = 1) [67]. Ten interventions [36,47,29,37,40,31,44,49,86,88] followed the micronutrient recommendations in the United Nations International Multiple Micronutrient Antenatal Preparation (UNIMMAP) guidelines [17] or an adaptation (Supplementary Table 2). Seven described providing advice on supplement consumption [38,34,36,29,33,39]. In addition, 2 studies mentioned that prescriptions were provided for counseling on supplement consumption [29,33], and others included additional resources like: *1*) iron drops for women with severe anemia [40], *2*) ferrous sulfate + FA twice daily for 3 mo and dewormers and prophylactic treatment for malaria [49], and *3*) additional IFA (*n* = 1) [30,39], or only FA (*n* = 1) [41]. Only 1 study reported the additional delivery of energy–protein supplements [37].

Supplements were delivered by a community health worker (CHW) (*n* = 7) [33,39,40,46,49,86], a doctor (*n* = 4) [36,47,53,85], a midwife (*n* = 6) [48,51,68,53,82,88], or a combination of providers including doctor–CHW, nurse–CHW, or doctor, nurse, and paramedic. Six studies did not report the provider.

In all instances, the delivery of the supplement and counseling on its use was provided individually; however, 1 study was delivered in a mixed format. Follow-up was conducted in-person for 18 of the studies [38,34,36,47,29,33,37,40,31,44,46,49,85,84,[86], [87], [88]], and in-person plus phone calls and text messages in 2 cases [30,39]. The intervention was delivered through home visits (*n* = 5) [33,39,40,44,86] in antenatal clinics (*n* = 3), community clinics (*n* = 1), or health centers (*n* = 2), or by combining various platforms, such as health centers and home visits, and community clinics with home visits (*n* = 3).

Regarding timing, 10 interventions commenced in the first trimester, 2 in the second trimester, and 8 between the first and second trimesters. The frequency of intervention delivery, whether for follow-up or supplement replenishment, was as follows: daily, biweekly, weekly, fortnightly, monthly, and at 3 routine prenatal appointments. In 13 studies, the supplementation lasted until birth. However, in 4 studies, the intervention was extended ≤12 wk, and in another 30 d, 90 d, and 6 mo, all during the postpartum period. Only 3 studies were tailored to consider their religious and cultural sensitivities, explaining procedures in local languages, and providing brief additional counseling on diet according to local food availability. All the details of the intervention are presented in Table 2.

Of the 20 studies included in this type of intervention, 17 [38,36,47,33,37,39,31,44,46,49,83,85,84,87,88] presented outcomes related to hemoglobin concentration in pregnant women. Among these, 44.4% [38,34,36,47,33,40,49,85] reported favorable and statistically significant impacts, with improvements in hemoglobin concentrations observed among women who received micronutrient tablets. In addition, half of those articles reported other indicators related to anemia, such as ferritin concentrations or percentages [33,52], abnormal ferritin serum concentrations [47], or anemia prevalence [34,36,33,44,49,85,84], and 66% showed improvements in these indicators. Moreover, in all the studies with positive outcomes, the improvements were statistically significant. Two studies show a reduction in hemoglobin concentrations compared with baseline measurements and with those taken in late pregnancy for the same indicators [32,33].

In studies where a favorable result was observed in hemoglobin concentrations or anemia indicators, supplementation was delivered individually, and the follow-up was conducted in-person. The latter occurred in most cases through doctors, CHWs, or a combination of both. Three of the studies were developed using the UNIMMAP formula. Two interventions were initiated in the first trimester of pregnancy, and 4 more were initiated between the first and second trimesters.

Finally, 2 studies centered their results on gestational weight gain. One of them reported that women who received multivitamin–mineral supplements had higher weight and BMI at 28 wk of pregnancy at delivery compared with those who only received multivitamins. The other study showed negative results for inadequate and adequate weight gain with micronutrient supplements, but positive results for adequate and excessive weight gain. One study that provided SQ-LNS presented positive results for inadequate weight gain, negative results for adequate weight gain, and no effect on excessive weight gain. Both supplements were provided by CHWs and nurses during the first and second trimesters of pregnancy, individually, with face-to-face and fortnightly follow-ups.

#### Gestational weight-gain monitoring

Regarding this type of intervention, we identified 2 studies [48,35]. One was conducted in an upper-middle-income country [48], and the other in a high-income country [35]. Both studies were performed individually, in a face-to-face format, between the first and second trimesters of pregnancy. They were not tailored to the sociocultural context or needs of the patients. One was a cohort focused solely on gestational weight gain assessed monthly by CHW, which did not rely on any behavioral change model or design guidelines. They used a card to record and monitor maternal weight during pregnancy. The other is an RCT based on Self-Regulatory Theory, incorporating the 2009 guidelines from the IOM for counseling and materials (brochures and manuals). It involved regular weighing and feedback by midwives through antenatal clinics during routine appointments.

Both studies, as implemented, showed positive but nonstatistically significant results for improved percentage of weight status in kg according to height and weeks of gestation [47], as well as the mean difference in the weight change (kg) during pregnancy following the 2009 IOM guidelines. However, one of them also reported unfavorable results regarding the proportion of women who gained weight within the IOM guidelines.

#### Healthy eating counseling

For this intervention, we identified 5 studies [53,55,45,81,82] that provided counseling on healthy eating; 4 also offered additional nutritional education focusing on promoting healthy eating habits and behavioral change. All studies for this intervention were conducted in low-income countries in the sub-Saharan Africa region, except for one, which was carried out in an upper-middle-income country from the Latin American and Caribbean region. Three of the studies were RCTs, and 2 were quasi-experimental with a control group (QEWC).

Two studies were based on behavioral change models of recommendations (the Health Belief Model and the Health Behavioral Change Consortium recommendations). All interventions were informed by evidence-based guidelines, including the World Health Organization’s recommendations on antenatal care, local health and nutrition guidelines, and the 2009 IOM guidelines on gestational weight gain.

Four studies included resources and materials to support intervention delivery, such as brochures, text messages, logbooks, pamphlets, MUAC measuring tapes, guides to provide nutritional education and counseling through several modules, role-play activities, photographs, drama lectures, and question-and-answer sessions. One study also included activities related to agricultural productivity and Water, Hygiene, and Sanitation services [45]. Four studies were implemented by midwives, nutritionists, and CHWs–nutritionists, and 1 study did not describe the personnel delivering the intervention.

Four out of the 5 studies were delivered individually, whereas 1 used a mixed approach—combining both group and individual sessions [81]. Regarding the delivery format, 2 studies employed face-to-face and virtual text messages, whereas 2 were in-person only, and 1 was based solely on virtual text messages.

Concerning the implementation frequency of counseling sessions, 1 study had monthly follow-ups, whereas 3 were delivered during routine appointments (2 studies included 3 sessions, but in 1 study, the number of sessions was not reported). In another study, sessions were scheduled every third day. In most interventions, counseling sessions lasted between 30 and 60 min. In 2 studies, participants received 18 text messages weekly throughout the study’s duration.

All studies were conducted through health centers: 1 in the second trimester, 2 in the first trimester, and 3 between the first and second trimesters of pregnancy. Four were tailored considering the local language, socioeconomic, and nutritional needs of the target population, and the availability and acceptability of local foods.

Although implementation heterogeneity was observed among studies, we identified positive results in all of them and found statistically significant results in 80% of the cases across diverse pregnancy indicators related to malnutrition in all its forms [53,55,45,81,82]. The findings included a lower prevalence of anemia in 2 studies [45,81], an improvement in hemoglobin concentration in 2 additional studies [53,45], and an enhancement in MUAC in 3 studies. In addition, favorable results regarding gestational weight gain were identified in 3 studies where the outcome was expressed in kg [55,45], as BMI or as the prevalence of undernutrition.

Most of the studies shared specific characteristics, such as being designed based on guidelines and applied in health centers up to the third trimester of pregnancy. Also, interventions included various materials and resources to facilitate counseling and were delivered by midwives, nutritionists, and/or CHWs.

#### Physical activity

One RCT [64] from a high-income country in Europe, focused on promoting physical activity, followed by broader support for a healthy lifestyle, based on the 2009 IOM guidelines [89]. The intervention was implemented in antenatal clinics, delivered monthly and biweekly, individually using videos, in-person sessions, and video calls with doctors, medical assistants, and midwives, lasting 10 to 60 min, and tailored considering their individual circumstances from the first trimester of pregnancy through to postpartum. Positive and statistically significant impacts were observed in the proportion of participants who met the IOM recommendation for total weight gain during pregnancy. The proportion of women who exceeded the recommended gestational weight gain according to the IOM guidelines was lower among those who received the intervention.

#### Combinations of interventions

A total of 25 studies examined various combinations of nutritional interventions. Fifteen studies combined counseling on healthy eating, physical activity, and weight monitoring, which also included measurements of weight and height, as well as self-monitoring [[41], [42], [43],^69^,^50^,^56^,^57^,^60^,^61^,^63^,^65^,^66^,^68^,^67^]. Ten studies were RCTs, 3 were QEWC, 2 were cohort studies, and 1 was a case-control study. Most of the studies in this category were conducted in high-income countries (78%) and in the Americas region (43%).

Ten studies [69,50,56,57,61,63,65,66,68] were based on behavioral change models, including the Parents as Teachers Model, a Behaviorally Grounded Model, the Model of Health Program Planning, Michie’s Behavior Change Techniques, the Centering Pregnancy Model, Social Cognitive Theory, the COACH Model (Commit, Omit, Add, Communicate, Honor your wellness), Centering Pregnancy, and Pender’s Health Promotion Model.

Also, 14 were based on guidelines [[41], [42], [43],^69^,^50^,^56^,^57^,^60^,^61^,^63^,^65^,^68^,^90^], with the most notable being the 2009 IOM guidelines (*n* = 9) [42,43,50,56,60,61,63,65,66,68], the 1990 IOM guidelines (*n* = 5), and other guidelines such as those issued by the American College of Sports Medicine in 1998 (*n* = 1). Twelve of these studies [42,43,69,50,56,57,60,63,65,68,90] incorporated resources or materials to support the delivery of the interventions, such as brochures, videos, hands-on activities, instructional digital video disks, notebooks to support goal setting for both diet and exercise, text messages, cognitive behavioral materials (the type was not specified, but it was reported that self-monitoring and troubleshooting strategies were used), a web-based app, websites, pedometers, weight-gain charts, manuals, workbooks, and videos. The interventions were provided by a single person (*n* = 6), either a CHW (*n* = 1), a midwife (*n* = 1), or a nutritionist (*n* = 4). The remaining interventions (*n* = 9) were delivered by a team, including doctors, nutritionists, nurses, midwives, physical therapists, assistants, and psychologists. In 10 studies, the intervention was carried out individually, whereas in 2 studies, it was delivered in group settings, and in 2 studies, a mixed approach was used. The intervention format was face-to-face in 10 studies, virtual in 2, and combined (in-person sessions and phone follow-ups) in 3.

Regarding the setting, 8 studies were conducted in antenatal clinics, 6 in health centers, and 1 through home visits. The interventions began in the first trimester (*n* = 6), between the first and second trimesters (*n* = 7), and in the second trimester (*n* = 2), and all were tailored considering the social needs, cultural context, the BMI or nutritional status, and including personalized plans that incorporate the patients’ stage of behavior change.

The implementation frequency varied, with 3 interventions delivered in weekly or biweekly sessions, monthly, during routine appointments (5–15 visits), and in 3 to 10 contact sessions, each lasting between 10 and 120 min. In addition, 1 study was conducted through 10 phone calls, each lasting ∼60 min. Regarding the total duration of the interventions, 11 studies lasted until delivery or the final weeks of pregnancy (36 or 37 wk), whereas other studies extended it into the postpartum period.

We identified that after the combined intervention—counseling on healthy eating, physical activity, and weight monitoring—80% (*n* = 12) of the studies reported positive impacts on achieving gestational weight gain according to the IOM recommendations (1990 and 2009) (*n* = 12) [41,42,43,69,50,56,60,63,66,68,67]. In addition, 1 study also reported a positive impact on hemoglobin. A common feature among the studies was that their designs were based on established guidelines and tailored to include materials and various resources that supported the delivery of interventions across diverse contexts. In the remaining 20% (*n* = 3), there was an increase in the proportion of women in the intervention group who gained gestational weight below or above the IOM recommendations guidelines.

Another combination of interventions identified was counseling on healthy eating along with gestational weight-gain monitoring, which included measurements of weight and height by health personnel and self-monitoring of weight by the participants in 3 studies [80,53,58, 2[70,53,58], 2 RCTs, and 1 cohort study. Only 1 study was from a high-income country, and 2 from upper- and middle-income countries. The majority (*n* = 2) of the studies were conducted in Latin America and the Caribbean. None of these studies was based on a behavioral change model, nor did they provide any resources or materials. Two studies adhered to the 2009 IOM guidelines, whereas 2 were delivered by a doctor or nutritionist.

All were conducted individually and in a face-to-face format. Two of the studies took place in antenatal clinics during the first trimester, whereas the third was conducted in health centers between the first and second trimesters of pregnancy. The implementation periodicity was heterogeneous, as follows: *1*) monthly (*n* = 1) [51], *2*) in 3 sessions (*n* = 1), and *3*) monthly, bimonthly, and weekly (*n* = 1). Two of the interventions were tailored to meet participants’ preferences and specific nutritional needs through individualized plans. The interventions lasted until 36 to 37 wk of gestation or until birth.

Two studies had positive impacts, and 75% had positive and statistically significant results for healthy pregnancy weight gain (≤12 kg) and weight gain above the recommended amount by the IOM. However, in another study, despite the highly frequent delivery and continuous follow-up, an increase was reported in the proportion of women with normal weight who gained less weight than recommended by the IOM guidelines.

The third combination of interventions included counseling on healthy eating and physical activity. Of the 3 RCTs identified [51,54,59], only 1 was based on a behavioral change model, specifically the Social Learning Theory. Two studies were designed following various guidelines, including: *1*) the 2009 IOM guidelines, *2*) dietary and physical activity recommendations from the American College of Obstetricians and Gynecologists and the United Kingdom National Health Service, and *3*) Healthy Start–Young Family Network recommendations. Two studies included brochures and text messages to support the counseling. All the studies in this group were from high-income countries.

The interventions were reported to be delivered by various health providers, including: *1*) a midwife, *2*) a doctor–midwife team, and *3*) health care providers. Two of these interventions were implemented individually, whereas 1 was delivered in a group format. In addition, 2 interventions were provided face-to-face, and only 1 used a mixed approach, combining in-person sessions with follow-up via text messages.

The implementation of these studies was also heterogeneous, taking place in health centers, antenatal clinics, and community health centers. Two of them began operations in the first and second trimesters of pregnancy, whereas another started between the first and third trimesters. The implementation frequency varied: *1*) during 8 routine appointments, *2*) in 6 sessions, and *3*) weekly. The duration of counseling or contact sessions with health care providers ranged from 10 to 60 min, with some sessions lasting ≤90 min. In addition, 2 of the studies were tailored to the sociocultural context, nutritional needs, and individualized goals and schedules of the participants.

In this group of studies, although some positive results were observed in gestational weight gain following IOM recommendations, the intervention effects were heterogeneous across subgroups stratified by IOM recommendations and weight categories. Specifically, no statistically significant differences were observed between the control and intervention groups.

One study, referred to as QEWC, was from a lower-middle-income group from Asia and involved multiple micronutrient supplements based on the UNIMMAP formula (1 tablet/d), along with counseling on nutrition and healthy eating provided by CHWs with a monthly follow-up and supplement replenishment. A specific behavioral change model did not guide this intervention. Still, it included additional resources, such as prescriptions with consumption instructions, deworming treatments, and a daily dose of 60 mg of iron combined with 400 μg of FA, administered over a 6-mo period. The intervention lasted from the first trimester until birth. The intervention had a positive and statistically significant effect on hemoglobin concentrations and mean BMI among participants in the intervention group.

The last intervention combination included physical activity counseling and gestational weight-gain monitoring (including measured weight and height, weight-gain monitoring by health personnel, and self-weight monitoring by participants), with only 1 QEWC study identified [62]. This study was based on the Transtheoretical Model and several guidelines, including the 2009 IOM Gestational Weight-Gain Guidelines, Physical Activity Guidelines, Exercise is Medicine Canada, Canada’s Food Guide, and *Healthy Pregnancy… Healthy Baby – A New Life*, an online prenatal guide published by the Government of New Brunswick. This intervention used resources and materials, including personalized prescriptions accompanied by recommendations on physical activity and brochures. It was delivered by doctors and nurses individually in a face-to-face format at antenatal clinics during the routine appointments from the first trimester until delivery. The intervention had a positive impact on weight gain as per the 2009 IOM guidelines.

#### RoB

**FIGURE 2:**
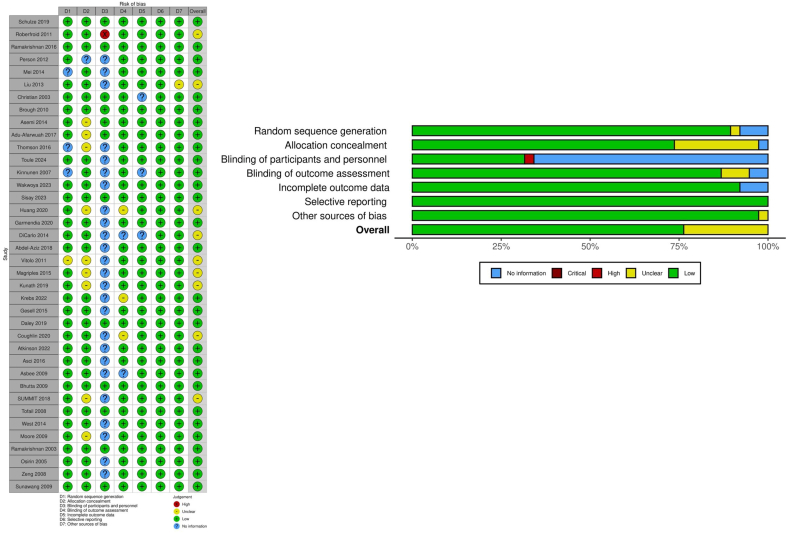
Risk of bias summary and graph for the randomized included studies using the Risk of Bias tool of the systematic review of effective implementation strategies for delivering nutritional interventions to prevent malnutrition during pregnancy.

Our analysis identified a low RoB in most of the studies**.** For the RCT design studies, 72% presented a low risk of overall bias, 24% an unclear risk of bias, and none a high risk of bias (Figure 2). We identified the participants’ domain blinding, which was not performed in most of the studies due to ethical considerations in vulnerable populations. The authors decided not to consider that domain for the overall risk of bias.

**FIGURE 3:**
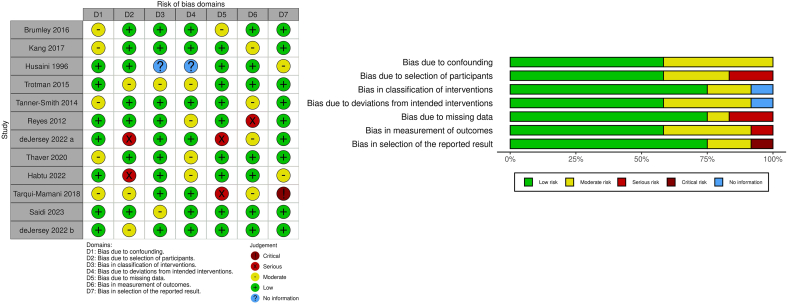
Risk of bias summary and graph for the nonrandomized included studies of the systematic review of effective implementation strategies for delivering nutritional interventions to prevent malnutrition during pregnancy.

For the nonrandomized studies, overall, they presented a moderate risk of bias (Figure 3). Because of the nature of the design of the studies, confounding factors were not always controlled for. However, these studies were identified as being of low-to-moderate concern. One study was identified as of critical concern [55] because the results presented in the tables were inconsistent with those in the text. A serious risk of bias concern was identified in another study due to missing data; the report presented unbalanced groups for the comparisons and reported a different precomparison group [50].

#### Meta-analysis

**FIGURE 4:**
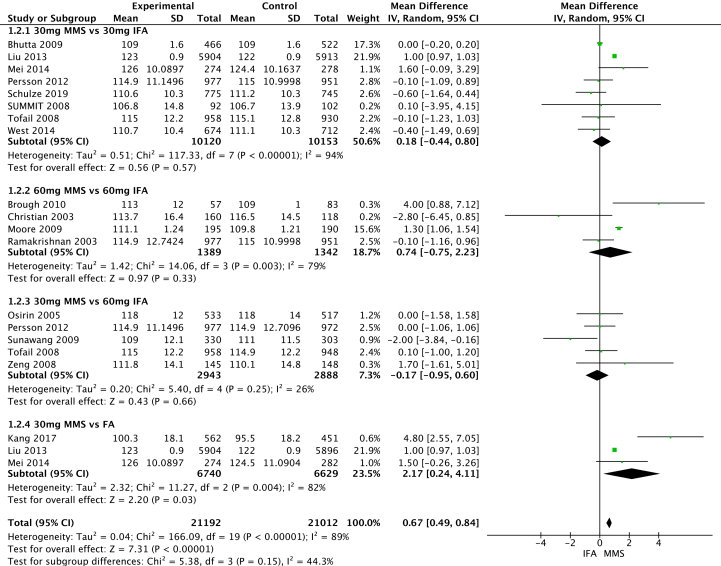
Forest plot of the mean difference of hemoglobin after the supplementation with multiple nutrients over the control group. CI, confidence interval; FA, folic acid; IFA, iron–folic acid supplementation; MMS, multiple micronutrient supplementation.

We identified 16 studies [38,34,36,47,33,37,40,31,44,46,83,85,88,91] with comparable data. In the pooled analysis of 20 intervention groups, a mean difference of 0.67 (95% CI: 0.49, 0.84) (Figure 4) in hemoglobin at the third trimester of pregnancy (range of 28–34 wk) was observed after supplementation with multiple nutrients in 21,192 women compared with the control group with moderate certainty of evidence (Table 4). This analysis presented an *I*^2^ of 89% overall. We conducted a subgroup analysis according to the dose of iron in the MMS and the iron given to the control group. For the group receiving 30 mg of iron in MMS compared with 30 mg of IFA, the mean difference was 0.18 (95% CI: –0.44, 0.80); for the group receiving 60 mg of iron in MMS compared with 60 mg of IFA, the mean difference was 0.74 (95% CI: –0.75, 2.23); for the group receiving 30 mg of iron in MMS and 60 mg of IFA, the mean difference was –0.17 (95% CI: –0.95, 0.60); and finally, for the group receiving 30 mg of iron in MMS compared with only FA, the mean difference was 2.17 (95% CI: 0.24, 4.11). After the leave-one-out sensitivity analysis, removing both groups from Liu et al. [36], the heterogeneity decreases in the subgroup of 30 mg of iron in MMS compared with 30 mg of iron in IFA to 0%. The sensitivity analysis conducted as part of this meta-analysis indicated robust and reliable findings.

**TABLE 4:** Summary of findings to evaluate the certainty of the evidence of hemoglobin after multiple micronutrient supplementation.

Certainty assessment							Summary of findings		
Participants (studies) Follow-up	Risk of bias	Inconsistency	Indirectness	Imprecision	Publication bias	Overall certainty of evidence	Study event rates (%)		Mean difference (95% CI)
							With FA	With multinutrient	
42,204 (16 RCTs)	Serious^1^	Not serious	Not serious	Not serious	None	⊕⊕⊕◯^1^ Moderate	21,012	21,192	Mean 0.67 mg/dL more (0.49 more to 0.84 more)

Abbreviations: CI, confidence interval; FA, folic acid; RCT, randomized controlled trial.

1 Downgraded because 8 studies were rated as unclear/no information for blinding of participants, and 5 studies showed unclear risk of bias for allocation concealment.

**FIGURE 5:**
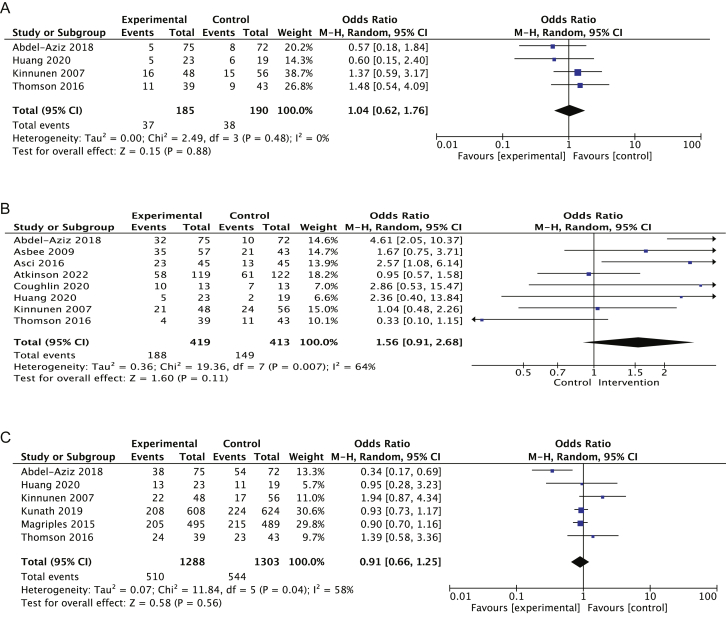
Forest plot of the prevalence of weight gain according to the IOM recommendation after a combined intervention of healthy eating and exercise counseling, and weight-gain management over the control group. (A) Forest plot of the prevalence of weight gain below the IOM recommendation. (B) Forest plot of the prevalence of weight gain within the IOM recommendation. (C) Forest plot of the prevalence of weight gain exceeding the IOM recommendation. CI, confidence interval; IOM, Institute of Medicine.

Regarding the prevalence of weight gain in relation to the IOM recommendation, the combined intervention of healthy eating and physical activity counseling, and weight-gain monitoring was compared with the control group. Six studies [56,57,60,61,65,67] with a total of 2591 participants identified a pooled OR of 0.91 (95% CI: 0.66, 1.25) with low certainty of the evidence of an excess of weight gained with low heterogeneity. In 8 studies [41,42,69,56,57,65,68,67,92] with a total of 832 participants, we identified a pooled OR of 1.56 (95% CI: 0.91, 2.68) of the prevalence within the recommendation with an *I*^2^ of 64% and very low certainty of the evidence. Four studies [56,57,65,67] identified a weight gain below the recommendation with an OR of 1.04 (95% CI: 0.62, 1.76) and very low certainty of the evidence (Figure 5; Table 5).

**TABLE 5:** Summary of findings to evaluate the certainty of the evidence of weight gain according to IOM recommendations.

Combined interventions compared with regular intervention for pregnancy nutrition outcomes											
Certainty assessment							Summary of findings				
Participants (studies) Follow-up	Risk of bias	Inconsistency	Indirectness	Imprecision	Other considerations	Overall certainty of evidence	Study event rates (%)		Relative effect (95% CI)	Anticipated absolute effects	
							With regular intervention	With combined interventions		Risk with regular intervention	Risk difference with combined interventions
Excessive weight gains according to IOM recommendations											
2591 (6 RCT)	Serious^1^	not serious	not serious	Serious^2^	None	⊕⊕◯◯ Low^1^^,^^2^	544/1303 (41.7)	510/1288 (39.6)	OR: 0.91 (0.66, 1.25)	417/1000	23 fewer per 1000 (from 62 fewer to 126 more)
Low weight gains according to IOM recommendations											
375 (4 RCT)	Serious^1^	Serious^3^	Not serious	Serious^4^	None	⊕⊕◯◯ Very low^1^^,^^3^^,^^4^	38/190 (20.0)	37/185 (20.0)	OR: 1.04 (0.62, 1.76)	200/1000	6 more per 1000 (from 66 fewer to 103 more)
Weight gain within the IOM recommendations											
832 (8 RCT)	Serious^1^	Serious^5^	Not serious	Serious^6^	None	⊕⊕◯◯ Very low^1^^,^^5^^,^^6^	149/413 (36.1)	188/419 (44.9)	OR: 1.56 (0.91, 2.68)	361/1000	107 more per 1000 (from 21 fewer to 241 more)

Abbreviations: CI, confidence interval; IOM, Institute of Medicine; OR, odds ratio; RCT, randomized controlled trial.

1 Downgraded because all studies presented unclear or no information on the blinding of participants and personnel. Four studies showed unclear risk of bias for allocation concealment. Two studies did not present information on random sequence generation.

2 Downgraded because the 95% CI is a wide crossing of the minimally important difference. The CI crosses the null and both decision thresholds for clinical significance.

3 Downgraded because there was substantial unexplained heterogeneity (*I*^2^ = 58%) across the 4 included studies.

4 Downgraded because of a null effect. The pooled OR of 1.04 (95% CI: 0.62, 1.76) is entirely compatible with no effect, a moderate benefit, or a moderate harm.

5 *I*^2^ = 64%, indicating substantial heterogeneity.

6 Downgraded because CI crosses the minimally important difference 0.10. Although the point estimate favors the combined intervention, the lower bound of the CI includes the possibility of harm.

The sensitivity analysis conducted as part of these meta-analyses indicated robust and reliable findings. The funnel plot analysis did not reveal any significant risk of publication bias. This result reinforces the validity of the combined intervention’s effects on weight gain in relation to the IOM recommendation.

## Discussion

The results of this systematic review show that delivering interventions in isolation does not yield comprehensive outcomes for preventing all forms of malnutrition that may affect pregnant women. Our meta-analysis also suggests that MMS for maternal anemia, compared with IFA supplements (20 intervention groups; mean difference: 0.67, 95% CI: 0.49, 0.84, with moderate-certainty evidence), increases hemoglobin concentrations. In addition, women who received a combination of nutritional counseling, physical activity, and weight-gain monitoring had higher odds of maintaining weight gain within the IOM recommendations and a lower risk of excessive weight gain. Prenatal care is a strategic platform for integrating nutritional interventions—not only to prevent maternal malnutrition but also to mitigate its short- and long-term consequences [3]. Global antenatal care guidelines emphasize the need to implement the studied nutritional interventions to prevent malnutrition and promote a healthy pregnancy [17,93].

Integrating nutritional interventions systematically into primary health care within health systems is essential for addressing malnutrition in pregnant women—particularly those experiencing multiple forms—and for improving health outcomes such as increased birthweight [94]. Vertical delivery of these intervention risks exacerbating nutritional inequities and associated burdens [13].

In our study, micronutrient supplementation strategies demonstrated positive outcomes, including improved hemoglobin concentrations and reduced anemia prevalence. However, addressing gestational weight gain remains critical, as pregnant women often experience inadequate or excessive weight gain. This requires reinforcing interventions that promote healthy eating, physical activity, and continuous weight-gain monitoring, while ensuring their feasibility in real-world settings.

This systematic review highlights effective ways to implement maternal nutrition interventions, allowing for adaptation to specific country contexts based on structural, multisectoral, and financial considerations. Such approaches not only improve coverage but also promote horizontal integration, ensuring vulnerable pregnant women receive timely, high-quality nutrition interventions [95].

Despite evidence supporting the effectiveness of prenatal micronutrient supplements, implementation remains challenging [93,96], particularly due to unclear guidelines for enhancing effectiveness through strategies like improved communication and counseling. Our systematic review identified only a few studies that reported whether counseling on supplement intake was provided, and none of them specified whether it was based on a behavioral change model. Researchers should provide detailed implementation frameworks to ensure that interventions are adaptable across diverse contexts while retaining their core functions [17,93]. This is crucial for offering clear guidance on how these interventions can be adapted across different contexts without sacrificing their core functions.

Most previous systematic reviews have focused on biological outcomes of micronutrient supplementation but lacked details on implementation strategies. Our review found that the best results in hemoglobin concentrations and anemia indicators occurred when supplementation began early in pregnancy, ideally between the first and second trimesters. Early initiation depends on factors such as access to prenatal counseling, education levels, urban residence, early pregnancy detection, socioeconomic status, and availability of trained health personnel [[97], [98], [99]].

Positive results in hemoglobin and anemia indicators were more commonly observed in studies involving in-person follow-up and counseling, as opposed to virtual follow-up methods like text messaging. These findings align with research by Gomes et al. [96], which showed no significant differences in hemoglobin concentrations with virtual follow-up.

Regarding SQ-LNS, its ability to provide both macro- and micronutrients makes it a good component of prenatal care, particularly in low- and middle-income countries where maternal undernutrition risks are higher. In the MMS group of studies, positive results were observed for inadequate gestational weight gain.

Our meta-analysis agrees with the evidence presented in the WHO updated recommendation on Multiple Micronutrient Supplements [17] and prior reviews. Our meta-analysis supports the WHO recommendation on MMS [100,101] and prior reviews, showing improved maternal anemia and hemoglobin concentrations, especially when comparing MMS to FA supplements, regardless of whether interventions used the UNIMMAP formula.

In our study, the most frequent combination of interventions included healthy eating counseling, physical activity, and weight-gain monitoring. These interventions showed variability in their implementation processes, delivery methods, and providers. However, they have previously been shown to reduce the risk of excessive gestational weight gain, as recommended by the IOM [99]. Our meta-analysis supports this conclusion, with pooled results indicating favorable outcomes, including maintaining weight gain within IOM recommendations and a reduced risk of excessive weight gain.

This underscores the importance of behavioral counseling, promoting physical activity, and monitoring gestational weight gain in achieving optimal pregnancy outcomes. We observed positive results in 80% of the included studies, with improvements across various indicators of malnutrition in all its forms, consistent with findings from previous reviews [96]. However, interventions delivered in isolation risk losing complementary benefits, such as supplementation to address micronutrient deficiencies [97], physical activity to reduce risks of preeclampsia and gestational diabetes, and weight monitoring to improve adherence to IOM recommendations [99].

We found that when delivered individually, positive results were identified regarding gestational weight gain. Harrison et al. [100] evaluated the delivery of behavioral counseling interventions for pregnant women with similar combined interventions; most were delivered in an individual and face-to-face format, with no perceptible differences in gestational weight gain during pregnancy.

Regarding the type of provider, no specific orientation toward a positive outcome was identified in the combined interventions. Healthy eating counseling interventions showed variability in implementation, often provided by midwives, nutritionists, and CHWs. Involving nonphysician personnel is critical for improving efficiency and distributing workload. Previously, O’Connor et al. [96] identified that interventions delivered by a nutrition professional were significantly more effective in improving diet quality.

Also, interventions promoting a healthy lifestyle during pregnancy were delivered mostly face-to-face. This and mixed delivery formats were more effective than virtual methods for promoting healthy lifestyles during pregnancy, as highlighted previously by Li et al. [102].

Although in our review, most of the studies were based on international prenatal care guidelines such as those from the IOM, and most of them identified positive results for weight gain, it is important to consider other aspects of these interventions, such as the health literacy of pregnant women and a deep understanding of how to foster behavior change in this population, to improve their effectiveness and sustainability.

Policy implications emphasize that maternal nutrition interventions should be formally integrated into routine prenatal care at the primary health care level, rather than implemented as isolated pilot projects. This aligns with global calls to institutionalize nutrition interventions within universal health coverage, incorporating them into funded and supervised maternal health service packages. This means that such interventions should be included as part of an explicit, funded, and supervised package of benefits within the essential maternal health services [13,101].

To ensure scalability, prenatal services require standardized models, clear implementation guidelines, defined roles for health personnel, and effective coordination mechanisms. Incorporating simple, low-cost counseling tools, as demonstrated in this study, can improve outcomes related to maternal malnutrition.

For sustainability, these interventions must be recognized as a vital component of maternal health and well-being, with stable funding allocated within national health plans. They should be integrated into all core health system components, including financing, a consistent supply of supplements, continuous training for health personnel, and a unified system for intervention tracking. From a governance perspective, policies should be formulated to support and promote their systematic integration into the health system.

### Strengths and limitations

Among the main strengths of this review is that it covers 4 essential types of interventions for preventing malnutrition in pregnant women, which are implemented through the health system at the primary health care during prenatal care. In addition, it highlights that the systematization of information was carried out considering key elements for the implementation of these interventions and to achieve better outcomes in some form of malnutrition in pregnant women, including the provider, materials, timing of intervention initiation, and its adaptation to the sociocultural context of the target population. It also highlights the importance of including studies from middle- and low-income countries, contributing to the gap in global guidelines regarding the need for research on the effects of these interventions across these contrasting contexts. Moreover, the interventions were examined both in isolation and in combination to assess the outcomes related to malnutrition in pregnant women in each case.

Regarding the limitations, it is essential to note that the information from some studies was not organized according to the TIDieR template; therefore, extracting certain components required assumptions from the reviewers. Furthermore, in some cases, there was incomplete information about the descriptions of the interventions included in the studies.

In conclusion, this systematic review and meta-analysis provides insights into some of the effective strategies for implementing essential nutritional interventions to prevent maternal malnutrition during pregnancy. These interventions should be delivered jointly at the primary health care to ensure that pregnant women and their offspring receive comprehensive benefits. Micronutrient supplementation interventions involving doctors or CHWs, which follow an individual delivery format and include in-person counseling, contribute to pregnancy-positive outcomes in hemoglobin concentrations and anemia-related indicators. When combined with healthy eating counseling, physical activity promotion, and gestational weight-gain monitoring adapted to the sociocultural, political, and health care system context and nutritional needs of pregnant women, it can help ensure appropriate gestational weight gain. The various implementation strategies identified can be adapted across different settings according to the available organizational structure and capacity.

## Author contributions

The authors’ responsibilities were as follows – SP-M, AB-A, AG-R, EK-H: involved in the conception and design of the manuscript; SP-M, AG-R, EK-H: independently screened titles, abstracts, and full-text articles; SP-M, AB-A, AG-R, EK-H, ED-G, LH-B: contributed to the draft of the manuscript; SP-M, AG-R, EK-H: carried out data extraction and analysis; and all authors: contributed to the interpretation of data, reviewed and approved the manuscript in its final version.

## Data availability

Data described in the manuscript will be made available upon request to the corresponding author.

## Funding

Funding for the research was provided by Vitamin Angels Alliance. Enrique Rios-Espinosa, a member of this organization, contributed to the review of the manuscript.

## Declaration of generative AI and AI-assisted technologies in the writing process

No artificial intelligence (AI) tools or generative AI technologies were used in the development, writing, or editing of this manuscript

## Conflict of interest

The authors report no conflicts of interest.
