# Supplementary material for: Effective Implementation Strategies for Delivering Nutritional Interventions through the Health System to Prevent Malnutrition during Pregnancy: A Systematic Review and Meta-analysis
Source: Adv Nutr. 2026 Apr 29;17(6):100642. doi: 10.1016/j.advnut.2026.100642 (PMC13223953; doi:10.1016/j.advnut.2026.100642)
Supplement: multimedia component 1 [file mmc1.pdf]

Supplementary material

"Effective implementation strategies for delivering nutritional interventions through the health system to prevent malnutrition during pregnancy: A Systematic Review and Meta-analysis."

First Author: Selene Pacheco Miranda

Supplementary Table 1. Detailed search strategy

Database	Search strategy
EMBASE	('pregnant woman'/exp OR 'pregnant woman' OR 'pregnancy'/exp OR 'pregnancy' OR 'expectant mother' OR 'gestation')<br>AND<br>('multiple micronutrient supplementation' OR 'maternal multiple micronutrient supplementation' OR 'micronutrient supplementation during pregnancy' OR 'multivitamin supplementation during pregnancy')<br>OR<br>('nutritional assessment'/exp OR 'weight monitoring'/exp OR 'weight gain'/exp OR 'nutritional assessment' OR 'weight monitoring' OR 'weight gain' OR 'nutrition evaluation')<br>OR<br>('healthy eating promotion' OR 'nutrition programs'/exp OR 'nutrition programs')<br>OR<br>('physical activity promotion' OR 'physical activity' OR 'sedentary' OR 'exercise')<br>AND

	('primary health care'/exp OR 'health service'/exp OR 'prenatal care'/exp OR 'antenatal care'/exp OR 'primary care' OR 'health service' OR 'prenatal care' OR 'antenatal care')
Science Direct	("pregnant woman" OR "pregnancy" OR "antenatal care" OR "prenatal care")<br>AND<br>("multiple micronutrient supplementation" OR "maternal multiple micronutrient supplementation" OR "micronutrient supplementation during pregnancy" OR "multivitamin supplementation during pregnancy")<br>OR<br>("nutritional assessment" OR "weight monitoring" OR "weight gain" OR "nutrition evaluation")<br>OR<br>('healthy eating promotion' OR 'nutrition programs')<br>OR<br>('physical activity promotion' OR 'physical activity' OR 'sedentary' OR exercise)<br>AND<br>("primary care" OR "health service" OR "prenatal care" OR "antenatal care")

Web of Science	TS=("pregnant woman" OR "pregnancy" OR "antenatal care" OR "prenatal care")<br>AND<br>TS=("multiple micronutrient supplementation" OR "maternal multiple micronutrient supplementation" OR "micronutrient supplementation during pregnancy" OR "multivitamin supplementation during pregnancy")<br>OR<br>TS=("nutritional assessment" OR "weight monitoring" OR "weight gain" OR "nutrition evaluation")<br>OR<br>(ALL=(healthy eating promotion) OR ALL=("nutrition programs") OR ALL=("nutrition guidance") OR ALL=("nutrition education")) OR ALL=(nutrition service))<br>OR<br>(ALL=("physical activity promotion") OR ALL=(sedentary) OR ALL=("exercise"))<br>AND<br>TS=("primary care" OR "health service" OR "prenatal care" OR "antenatal care")

Cochrane reviews	("pregnant woman" OR "pregnancy" OR "antenatal care" OR "prenatal care")<br>AND<br>("multiple micronutrient supplementation" OR "maternal multiple micronutrient supplementation" OR "micronutrient supplementation during pregnancy" OR "multivitamin supplementation during pregnancy")<br>OR<br>("nutritional assessment" OR "weight monitoring" OR "weight gain" OR "nutrition evaluation")<br>OR<br>(“nutrition education” OR “nutrition program” OR “nutrition counseling”)<br>AND<br>("primary care" OR "health service" OR "prenatal care" OR "antenatal care")
PubMed	("pregnant woman"[MeSH Terms] OR "pregnant woman" OR "pregnancy"[MeSH Terms] OR "pregnancy")<br>AND

	("multiple micronutrient supplementation" OR "maternal multiple micronutrient supplementation" OR "micronutrient supplementation during pregnancy" OR "multivitamin supplementation during pregnancy")<br>OR<br>("nutritional assessment"[Title/Abstract] OR "weight monitoring"[Title/Abstract] OR "weight gain"[Title/Abstract] OR "nutrition evaluation"[Title/Abstract])<br>OR<br>(("physical activity promotion"[Title/Abstract] OR "physical activity"[Title/Abstract] OR "sedentary"[Title/Abstract] OR "exercise"[Title/Abstract] OR "fitness"[Title/Abstract] OR "active lifestyle"[Title/Abstract] OR "movement"[Title/Abstract]))<br>AND<br>("primary care"[Title/Abstract] OR "health service"[Title/Abstract] OR "prenatal care"[Title/Abstract] OR "antenatal care"[Title/Abstract] OR "antenatal care"[MeSH Terms] OR "antenatal care" OR "prenatal care"[MeSH Terms] OR "prenatal care")

Supplementary Table 2. Supplement formulation used in the supplementation intervention group

Author and year	Supplement formulation
Thaver 2020	**UNIMMAP**<br>Vitamin A (800 µg/retinol equivalents), Vitamin D (200 IU), Vitamin E (10 mg), Vitamin C (70 mg), Thiamin (1.4 mg), Riboflavin (1.4 mg), Niacin (18 mg), Vitamin B6 (1.9 mg), Folic Acid (400 µg), Vitamin B12 (2.6 µg), Cooper (2 mg), Iodine (150 µg), Iron (30 mg as iron fumarate or iron sulphate), Selenium (65 µg), Zinc (15 mg)
Schulze 2019	Vitamin A (770 µg retinol activity equivalents), Vitamin D (5 µg, 200 IU), Vitamin E (15 mg), Vitamin B-1 (thiamin, 1.4 mg), Vitamin B-2 (riboflavin, 1.4 mg), Vitamin B-3 (niacin, 18 mg), Vitamin B-6 (1.9 mg), Vitamin B-12 (2.6 µg), and Vitamin C (85 mg) and Zinc (12 mg), Copper (1 mg), Selenium (60 µg), and Iodine (220 µg), Iron (27mg), and Folic Acid (600 µg)
Roberfroid 2011	**UNIMMAP**

	Vitamin A (800 µg/retinol equivalents), Vitamin D (200 IU), Vitamin E (10 mg), Vitamin C (70 mg), Thiamin (1.4 mg), Riboflavin (1.4 mg), Niacin (18 mg), Vitamin B6 (1.9 mg), Folic Acid (400 µg), Vitamin B12 (2.6 µg), Cooper (2 mg), Iodine (150 µg), Iron (30 mg), Selenium (65 µg), Zinc (15 mg)
Ramakrishnan 2016	**MM group (multiple micronutrients):**<br>Vitamin A (800 µg), Vitamin D (600 IU), Vitamin E (10 mg), Vitamin C (70 mg), Thiamin (1.4 mg), Riboflavin (1.4 mg), Niacin (18 mg), Vitamin B-6 (1.9 mg), Vitamin B-12 (2.6 µg), Folic acid (2800 µg), Iron as ferrous sulfate (60 mg), Zinc as sulfate (15 mg), Copper (2 mg), Selenium (65 µg), and Iodine (150 µg).<br><br>**IFA group (Iron and Folic Acid):**<br>Folic Acid (2800 µg) and Iron as ferrous sulfate (60 mg).<br><br>**FA group (Folic Acid only):**<br>Folic Acid (2800 µg).
Persson 2012	**Group 1**

	Iron (fumarate) (30 mg) and Folic Acid (400 µg)<br><br>**Group 2**<br>Iron (fumarate) (60 mg) and Folic Acid (400 µg)<br><br>**Group 3 UNIMMAP**<br>Vitamin A (800 µg/retinol equivalents), Vitamin D (200 IU), Vitamin E (10 mg), Vitamin C (70 mg), Thiamin (1.4 mg), Riboflavin (1.4 mg), Niacin (18 mg), Vitamin B6 (1.9 mg), Folic Acid (400 µg), Vitamin B12 (2.6 µg), Cooper (2 mg), Iodine (150 µg), Iron (30 mg), Selenium (65 µg), Zinc (15 mg)
Mei 2014	**Group 1**<br>Folic Acid (400 µg)<br><br>**Group 2**<br>Iron (30 mg) and Folic Acid (400 µg)

	**Group 3 UNIMMAP**<br>Vitamin A (800 µg/retinol equivalents), Vitamin D (200 IU), Vitamin E (10 mg), Vitamin C (70 mg), Thiamin (1.4 mg), Riboflavin (1.4 mg), Niacin (18 mg), Vitamin B6 (1.9 mg), Folic Acid (400 µg), Vitamin B12 (2.6 µg), Cooper (2 mg), Iodine (150 µg), Iron (30 mg), Selenium (65 µg), Zinc (15 mg)
Liu 2013	**Group 1**<br>Folic Acid (400 µg)<br><br>**Group 2**<br>Iron (30 mg) and Folic Acid (400 µg)<br><br>**Group 3 UNIMMAP**

	Vitamin A (800 μg/retinol equivalents), Vitamin D (200 IU), Vitamin E (10 mg), Vitamin C (70 mg), Thiamin (1.4 mg), Riboflavin (1.4 mg), Niacin (18 mg), Vitamin B6 (1.9 mg), Folic Acid (400 μg), Vitamin B12 (2.6 μg), Cooper (2 mg), Iodine (150 μg), Iron (30 mg), Selenium (65 μg), Zinc (15 mg)
Kang 2017	**Materna/tablet; Wyeth Pharmaceutical Co., Ltd**<br>Vitamin A (450 μg), β-carotene (900 μg), Vitamin D (6.25 μg), Vitamin E (30 mg), Vitamin B1 (3 mg), Vitamin B2 (3.4 mg), Vitamin B6 (10 mg), Vitamin B12 (12 μg), Vitamin C (100 mg), Biotin (30 μg), Folic Acid (1 mg), Nicotinamide (20 mg), Pantothenic acid (10 mg), Iodine (150 μg), Molybdenum (25 μg), Calcium (250 mg), Zinc (25 mg), Iron (60 mg), Copper (2.0 mg), Chromium (25 μg), Manganese (5 mg), Magnesium (50 mg), and Selenium (25 μg).<br><br>**The folate-only supplement (Scrianen/tablet; Peking University Pharmaceutical Co., Ltd)**<br>Folic Acid (0.4 mg)
Christian 2003	**Group 1**<br>Vitamin A (1000 μg RE as retinol acetate)

	**Group 2**<br>Folic Acid (400 µg)<br><br>**Group 3**<br>Folic Acid (400 µg), Iron (60 mg as ferrous fumarate)<br><br>**Group 4**<br>Folic Acid (400 µg), Iron (60 mg as ferrous fumarate), Zinc (30 mg as zinc sulfate)<br><br>**Group 5**<br>Folic Acid (400 µg), Iron (60 mg as ferrous fumarate), Zinc (30 mg as zinc sulfate), Vitamin D (10 µg as D3), Vitamin E (10 mg as d-alpha tocopherol), Thiamine (1.6 mg), Riboflavin (1.8 mg), Niacin (20 mg), Vitamin B-6 (2.2 mg), Vitamin B-12 (2.6 µg), Vitamin C (100 mg), Vitamin K (65 µg as K1), Copper (2.0 mg), Magnesium (100 mg).

Brough 2010	**Pregnacare**<br>β-Carotene (3 mg), Thiamin (3 mg as thiamin mononitrate, 3.6 mg), Riboflavin (2 mg), Niacin (20 mg as nicotinamide), Vitamin B6 (10 mg as pyridoxine HCl), Vitamin B12 (6 μg as cyanocobalamin), Folic Acid (400 μg), Vitamin C (70 mg as ascorbic acid, 73 mg), Vitamin D (5 μg as cholecalciferol, 200 IU), Vitamin E (20 mg as D-α-tocopheryl acid succinate, 21 mg), Vitamin K (70 μg)<br>Iron (20 mg as ferrous fumarate, 63.3 mg), Zinc (15 mg as zinc sulfate H_2O, 41 mg), Magnesium (150 mg as magnesium hydroxide, 372 mg), Iodine (140 μg as potassium iodide, 183 μg), Copper (1 mg as copper sulfate H_2O, 2.8 mg)
Asemi 2014	**Multivitamin supplement:**<br>Vitamin A (5000 IU), Vitamin E (15 IU), Vitamin D3 (400 IU), Vitamin B1 (1.5 mg), Vitamin B2 (1.7 mg), Niacin (20 mg), Vitamin B6 (2 mg), Folic Acid (400 μg), Vitamin B12 (6 μg), Vitamin C (60 mg)<br><br>**Multivitamin-mineral supplement:**

	Vitamin A (4000 IU), Vitamin E (11 IU), Vitamin D3 (400 IU), Vitamin B1 (1.5 mg), Vitamin B2 (1.8 mg), Niacin (18 mg), Vitamin B6 (2.8 mg), Folic Acid (800 µg), Vitamin B12 (4 µg), Vitamin C (100 mg), Calcium (250 mg), Iron (27 mg), Zinc (25 mg)
Adu-Afarwuah 2017	**Group 1**<br>Folic Acid (400 µg), Iron (60 mg)<br><br>**Lipid-based Nutrient Supplements (LNS) group**<br>SQ-LNS (20 g), Linoleic Acid (4.59 g), α-Linolenic Acid (0.59 g), Vitamin A (800 µg RE), Vitamin C (100 mg), Vitamin B1 (2.8 mg), Vitamin B2 (2.8 mg), Niacin (36 mg), Folic Acid (400 µg), Pantothenic Acid (7 mg), Vitamin B6 (3.8 mg), Vitamin B12 (5.2 µg), Vitamin D (400 IU), Vitamin E (20 mg), Vitamin K (45 µg), Iron (20 mg), Zinc (30 mg), Copper (4 mg), Calcium (280 mg), Phosphorus (190 mg), Potassium (200 mg), Magnesium (65 mg), Selenium (130 µg), Iodine (250 µg), Manganese (2.6 mg)<br><br>**MMN supplement group:**

	Vitamin A (800 µg RE), Vitamin C (100 mg), Vitamin B1 (2.8 mg), Vitamin B2 (2.8 mg), Niacin (36 mg), Folic Acid (400 µg), Pantothenic Acid (7 mg), Vitamin B6 (3.8 mg), Vitamin B12 (5.2 µg), Vitamin D (400 IU), Vitamin E (20 mg), Vitamin K (45 µg), Iron (20 mg), Zinc (30 mg), Copper (4 mg), Selenium (130 µg), Iodine (250 µg), Manganese (2.6 mg)
Ramakrishnan 2004	Vitamin A (2150 IU), Vitamin D_3 (309 IU), Vitamin E (5.73 IU), Thiamine (0.93 mg), Riboflavin (1.87 mg), Niacin (15.5 mg), Folic acid (215 µg), Vitamin B-6 (1.94 mg), Vitamin B-12 (2.04 µg), Vitamin C (66.5 mg), Zinc (12.9 mg), Iron (62.4 mg) y Magnesium (252 mg).
Vaidya 2008	Vitamin A (800 µg), Vitamin E (10 mg), Vitamin D (5 µg), Vitamin B1 (1.4 mg), Vitamin B2 (1.4 mg), Niacin (18 mg), Vitamin B6 (1.9 mg), Vitamin B12 (2.6 µg), Folic acid (400 µg), Vitamin C (70 mg), Iron (30 mg), Zinc (15 mg), Copper (2 mg), Selenium (65 µg) y Iodine (150 µg).
SUMMIT Study Group	**UNIMMAP**<br>Vitamin A (800 µg/retinol equivalents), Vitamin D (200 IU), Vitamin E (10 mg), Vitamin C (70 mg), Thiamin (1.4 mg), Riboflavin (1.4 mg), Niacin (18 mg), Vitamin B6 (1.9 mg), Folic Acid (400 µg), Vitamin B12 (2.6 µg), Cooper (2 mg), Iodine (150 µg), Iron (30 mg), Selenium (65 µg), Zinc (15 mg).

Tofail 2008	**MMN Group**<br>Vitamin A (800 µg), Vitamin E (10 mg), Vitamin D (5 µg), Vitamin B1 (1.4 mg), Vitamin B2 (1.4 mg), Niacin (18 mg), Vitamin B6 (1.9 mg), Vitamin B12 (2.6 µg), Folic Acid (400 µg), Vitamin C (70 mg), Iron (30 mg), Zinc (15 mg), Copper (2 mg), Selenium (65 µg), and Iodine (150 µg).<br>**Fe60 group**<br>Iron (Fumarate) (60 mg) + Folate (400 μg).<br>**Fe30 group**<br>Iron (Fumarate) (30 mg) + Folate (400 μg).
Sunawang 2009	**UNIMMAP**<br>Vitamin A (800 µg/retinol equivalents), Vitamin D (200 IU), Vitamin E (10 mg), Vitamin C (70 mg), Thiamin (1.4 mg), Riboflavin (1.4 mg), Niacin (18 mg), Vitamin B6 (1.9 mg), Folic Acid (400 µg), Vitamin B12 (2.6 µg), Cooper (2 mg), Iodine (150 µg), Iron (30 mg), Selenium (65 µg), Zinc (15 mg).
West 2014	**UNIMMAP**

	Vitamin A (800 µg/retinol equivalents), Vitamin D (200 IU), Vitamin E (10 mg), Vitamin C (70 mg), Thiamin (1.4 mg), Riboflavin (1.4 mg), Niacin (18 mg), Vitamin B6 (1.9 mg), Folic Acid (400 µg), Vitamin B12 (2.6 µg), Cooper (2 mg), Iodine (150 µg), Iron (30 mg), Selenium (65 µg), Zinc (15 mg).
Moore 2019	**FeFol group**<br>Iron (60 mg), Folate (400 µg).<br>**MMN group**<br>Iron (60 mg), Folate (400 µg), Vitamin A (1600 µg RE), Vitamin D (400 IU), Vitamin E (20 mg), Vitamin C (140 mg), Vitamin B1 (2.8 mg), Vitamin B2 (2.8 mg), Niacin (36 mg), Vitamin B6 (2.8 mg), Vitamin B12 (5.2 µg), Zinc (30 mg), Copper (4 mg), Selenium (130 µg), Iodine (300 µg).<br>**PE+FeFol group**<br>Vitamin A (2.85 µg RE), Vitamin E (4.2 mg), Vitamin C (2.25 mg), Vitamin B1 (0.3 mg), Vitamin B2 (0.45 mg), Niacin (1.35 mg), Vitamin B6 (0.15 mg), Vitamin B12 (0.1 µg), Zinc (3.3 mg), Copper (1.05 mg), Selenium (6.15 µg), Iodine (2.6 µg), Energy (746 kcal), Protein (20.8 g), Lipids (52.6 g)<br>**PE+MMN group**

	Vitamin A (1600 μg RE), Vitamin D (400 IU), Vitamin E (20 mg), Vitamin C (140 mg), Vitamin B1 (2.8 mg), Vitamin B2 (2.8 mg), Niacin (36 mg), Vitamin B6 (2.8 mg), Vitamin B12 (5.2 μg), Zinc (30 mg), Copper (4 mg), Selenium (130 μg), Iodine (300 μg), Energy (746 kcal), Protein (20.8 g), Lipids (52.6 g)
Zeng 2008	**UNIMMAP**<br>Vitamin A (800 μg/retinol equivalents), Vitamin D (200 IU), Vitamin E (10 mg), Vitamin C (70 mg), Thiamin (1.4 mg), Riboflavin (1.4 mg), Niacin (18 mg), Vitamin B6 (1.9 mg), Folic Acid (400 μg), Vitamin B12 (2.6 μg), Cooper (2 mg), Iodine (150 μg), Iron (30 mg), Selenium (65 μg), Zinc (15 mg).
Buttha 2009	**UNIMMAP**<br>Vitamin A (800 μg/retinol equivalents), Vitamin D (200 IU), Vitamin E (10 mg), Vitamin C (70 mg), Thiamin (1.4 mg), Riboflavin (1.4 mg), Niacin (18 mg), Vitamin B6 (1.9 mg), Folic Acid (400 μg), Vitamin B12 (2.6 μg), Cooper (2 mg), Iodine (150 μg), Iron (30 mg), Selenium (65 μg), Zinc (15 mg).

UNIMMAP: United Nations International Multiple Micronutrient Antenatal Preparation Multiple Micronutrient Supplements; IU: International Units; SQ-LNS: Small-quantity Lipid-based Nutrient Supplements; LNS, lipid-based nutrient supplement; MMN, multiple micronutrient supplement; FeFol, iron folic acid; PE, protein energy.
